# N^6^‐Methyladenosine Demethylase FTO Contributes to Neuropathic Pain by Stabilizing G9a Expression in Primary Sensory Neurons

**DOI:** 10.1002/advs.201902402

**Published:** 2020-05-27

**Authors:** Yize Li, Xinying Guo, Linlin Sun, Jifang Xiao, Songxue Su, Shibin Du, Zhen Li, Shaogen Wu, Weili Liu, Kai Mo, Shangzhou Xia, Yun‐Juan Chang, Daniel Denis, Yuan‐Xiang Tao

**Affiliations:** ^1^ Department of Anesthesiology New Jersey Medical School, Rutgers The State University of New Jersey 185 S. Orange Ave., MSB E594 Newark NJ 07103 USA; ^2^ Department of Physiology, Pharmacology & Neuroscience New Jersey Medical School, Rutgers The State University of New Jersey 185 S. Orange Ave., MSB E661 Newark NJ 07103 USA; ^3^ The Office of Advanced Research Computing Rutgers, The State University of New Jersey 185 S. Orange Ave., MSB C‐630 Newark NJ 07103 USA; ^4^ Department of Cell Biology & Molecular Medicine New Jersey Medical School, Rutgers The State University of New Jersey 185 S. Orange Ave., MSB E661 Newark NJ 07103 USA

**Keywords:** dorsal root ganglion, euchromatic histone lysine methyltransferase 2, fat‐mass and obesity‐associated proteins, histone methyltransferase G9a, m^6^A modification, neuropathic pain

## Abstract

Nerve injury‐induced change in gene expression in primary sensory neurons of dorsal root ganglion (DRG) is critical for neuropathic pain genesis. N^6^‐methyladenosine (m^6^A) modification of RNA represents an additional layer of gene regulation. Here, it is reported that peripheral nerve injury increases the expression of the m^6^A demethylase fat‐mass and obesity‐associated proteins (FTO) in the injured DRG via the activation of Runx1, a transcription factor that binds to the *Fto* gene promoter. Mimicking this increase erases m^6^A in euchromatic histone lysine methyltransferase 2 (*Ehmt2*) mRNA (encoding the histone methyltransferase G9a) and elevates the level of G9a in DRG and leads to neuropathic pain symptoms. Conversely, blocking this increase reverses a loss of m^6^A sites in *Ehmt2* mRNA and destabilizes the nerve injury‐induced G9a upregulation in the injured DRG and alleviates nerve injury‐associated pain hypersensitivities. FTO contributes to neuropathic pain likely through stabilizing nerve injury‐induced upregulation of G9a, a neuropathic pain initiator, in primary sensory neurons.

## Graphical abstract

This study explores the m^6^A epitranscriptional mechanism of neuropathic pain. Peripheral nerve injury produces the Runx1‐triggered FTO upregulation in the dorsal root ganglion (DRG). This upregulation contributes to nerve injury‐induced pain hypersensitivity through erasing *Ehmt2* mRNA m^6^A, stabilizing *Ehmt2* mRNA/G9a expression, and silencing mu opioid receptor expression in the injured DRG. FTO may be a new target for neuropathic pain treatment.

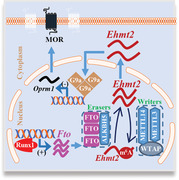

## 1 Introduction

Nerve injury‐induced neuropathic pain is a chronic, refractory disease that affects more than 4 million people in the United States alone.^[^ ^1^ ^]^ Therapeutic management for this disorder is limited in success as current medications such as opioids and nonsteroidal anti‐inflammatory drugs are ineffective and/or produce severe side effects in most neuropathic pain patients.^[^ ^2^ ^]^ Peripheral nerve injury leads to changes in the expression of pain‐associated genes at both the transcriptional and translational levels in the first‐order sensory neurons of dorsal root ganglia (DRG).^[^ ^3^, ^4^, ^5^ ^]^ These changes contribute to neuropathic pain development and maintenance.^[^ ^3^, ^6^, ^7^, ^8^ ^]^ Understanding of how these pain‐associated genes are altered in the DRG following peripheral nerve injury may provide a new potential avenue in neuropathic pain management.

G9a, encoded by euchromatic histone lysine methyltransferase 2 (*Ehmt2*) mRNA, is a repressor of gene transcription through its dimethylating histone H3 at Lys^9^ and subsequently condensing chromatin.^[^ ^9^, ^10^ ^]^ Peripheral nerve injury increases the expression of both *Ehmt2* mRNA and G9a in the injured DRG.^[^ ^3^, ^11^, ^12^, ^13^, ^14^ ^]^ These increases participated in nerve injury‐induced downregulation of opioid receptor‐coding genes and several potassium channel‐encoding genes in the injured DRG.^[^ ^3^, ^11^, ^12^, ^13^, ^14^ ^]^ Pharmacological inhibition or genetic knockout/knockdown of DRG G9a reduced DRG neuronal hyper‐excitability, diminished pain hypersensitivity, rescued opioid analgesia, and prevented opioid analgesic tolerance development under neuropathic pain conditions.^[^ ^3^, ^11^, ^12^, ^13^, ^14^ ^]^ G9a likely is an endogenous initiator in neuropathic pain. However, how *Ehmt2* mRNA and its coding G9a are increased in the DRG after peripheral nerve injury is incompletely understood.

N^6^‐methyladenosine (m^6^A) is the most prevalent internal modification found in at least one‐fourth of mammalian mRNAs, which is located typically in a consensus motif of DRACH (D = A, G, or U; R = A or G; H = A, U, or C) and enriched particularly around the transcription start site and at the beginning of the 3′‐UTR near the stop codons.^[^ ^15^, ^16^, ^17^ ^]^ m^6^A is installed by a multi‐subunit methyltransferase complex, including the methyltransferase‐like 3 and 14 (METTL3 and METTL14) and Wilms’ tumor 1‐associating protein (WTAP) and erased by at least two specific demethylases, fat‐mass and obesity‐associated proteins (FTO) and AlkB homolog 5 (ALKBH5).^[^ ^15^, ^16^, ^18^, ^19^ ^]^ This modification recruits diverse m^6^A‐binding proteins such as YTH N^6^‐methyladenosine RNA binding proteins1/2/3 (YTHDF1/2/3)^[^ ^15^, ^16^, ^18^ ^]^ to impact almost all stages of mRNA biogenesis, including RNA transcription, splicing, export, translation, and degradation.^[^ ^20^, ^21^, ^22^, ^23^, ^24^ ^]^ RNA m^6^A modification likely represents an additional layer of gene regulation. It is therefore not surprising that the methyltransferases/demethylases‐induced dysregulation of m^6^A RNA modification and the expressional changes of m^6^A‐binding proteins result in several physiological defects and participates in pathological processes in the nervous system.^[^ ^25^, ^26^, ^27^, ^28^, ^29^ ^]^ However, the role of m^6^A RNA modification in neuropathic pain is still elusive.

We report here that peripheral nerve injury leads to a significant increase in FTO, but not in METTL3, METTL14, ALKBH5, WTAP, and YTHDF2, in the injured DRG. This increase contributes to nerve injury‐induced neuropathic pain induction and maintenance at least in part through erasing the m^6^A in *Ehmt2* mRNA and stabilizing the nerve injury‐induced *Ehmt2* mRNA/G9a increase in the injured DRG. FTO is likely a potential new target for neuropathic pain management.

## 2 Results

### 2.1 FTO Is Increased in the Ipsilateral DRG after Peripheral Nerve Injury

**Figure 1:**
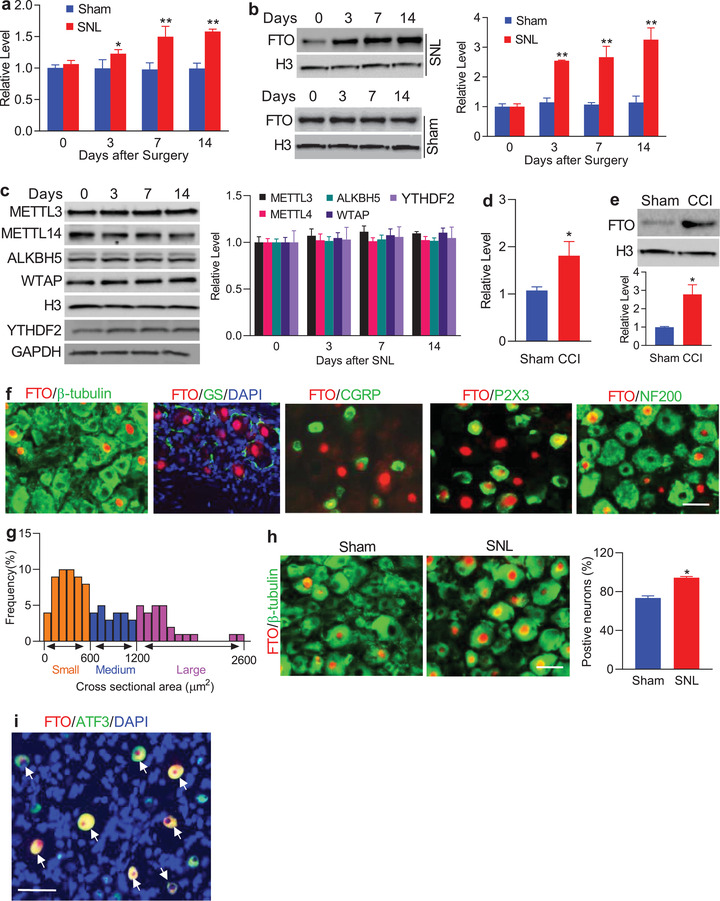
Nerve injury‐induced increases in the levels of *Fto* mRNA and FTO protein in the injured DRG. a,b) *Fto* mRNA (a) and FTO protein (b) expression in the ipsilateral L5 DRG after SNL or sham surgery; *n* = 6 rats/group/time point. *p < 0.05, **p < 0.01 versus the corresponding control group (0 day) by two‐way ANOVA with repeated measures followed by post hoc Tukey test. c) Expression of METTL3, METTLE14, ALKBH5, WTAP, and YTHDF2 in the ipsilateral L5 DRG after SNL; *n* = 6 rats/group/time point. One‐way ANOVA with repeated measures followed by post hoc Tukey test. d,e) *Fto* mRNA (d) and FTO protein (e) expression in the ipsilateral L4/5 DRGs on day 7 after CCI or sham surgery; *n* = 6 rats/group. *p < 0.05 versus the sham group by two‐tailed unpaired Student's *t*‐test. f) FTO (red) is co‐expressed with *β*‐tubulin III (green) in individual cells and undetected in cellular nuclei (labeled by 4′, 6‐diamidino‐2‐phenylindole (DAPI), blue) of glutamine synthetase (GS, green)‐labeled cells. Some FTO‐positive neurons were labeled by calcitonin gene‐related peptide (CGRP, green), P2X3 (green) or neurofilament‐200 (NF200, green); *n* = 5 rats (biological repeats). Scale bar: 40 µm. g) Histogram shows the distribution of FTO‐positive somata in normal rat L5 DRG: small, 50%; medium, 24%; large, 26%. h) Number of the neurons labeled by FTO (red) and *β*‐tubulin III (green) in the ipsilateral L5 DRG on day 7 after SNL or sham surgery; *n* = 5 rats/group. *p < 0.05 versus the sham group by two‐tailed unpaired Student's *t*‐test. Scale bar: 40 µm. i) Neurons (arrows) were labeled for FTO (red), ATF3 (green), and DAPI (blue) in the ipsilateral L5 DRG on day 7 post‐SNL; *n* = 5 rats. Scale bar: 40 µm.

To examine the role of DRG RNA m^6^A modification in neuropathic pain, we first analyzed the expression of methyltransferases and associated proteins, demethylases, and the m^6^A‐binding proteins in the DRG after the fifth lumbar (L5) spinal nerve ligation (SNL) in rats, a preclinical animal model that mimics nerve trauma‐induced neuropathic pain in clinical cases.^[^ ^30^ ^]^ Unilateral SNL increased the expression of *Fto* mRNA and FTO protein in a time‐dependent manner (**Figure** **1**a,b), but not METTL3, METTL14, WTAP, and YTHDF2 (Figure 1c), in the ipsilateral L5 DRG. None of these proteins displayed the changes in the contralateral L5 DRG and the ipsilateral L4 (intact) DRG (Figure S1a, Supporting Information). Results were similar after chronic constriction injury (CCI) of the sciatic nerve (Figure 1d,e), another preclinical animal model of neuropathic pain.^[^ ^31^ ^]^ Interestingly, the level of FTO protein was not significantly changed in the ipsilateral L4/5 DRGs from 2 h to 7 days after plantar injection of complete Freund's adjuvant (CFA) into unilateral hindpaw (Figure S1b, Supporting Information), a preclinical animal model of chronic inflammatory pain.^[^ ^32^ ^]^ DRG FTO expression in a specific response to peripheral nerve injury suggests a potential role of FTO in neuropathic pain.

The distribution pattern of FTO in the DRG was also examined. We carried out double/triple labeling assays and found that FTO co‐existed with *β*‐tubulin III (a specific neuronal marker) in individual cells (Figure 1f) and was not detected in the cellular nuclei (labeled by 4′, 6‐diamidino‐2‐phenylindole [DAPI]) of glutamine synthetase (GS, a marker for satellite glial cells)‐labeled cells (Figure 1f), indicating that FTO is expressed predominantly in DRG neurons. Approximately 73% of *β*‐tubulin III‐labeled neurons (402/548) were positive for FTO. Moreover, about 18% of FTO‐positive neurons were labeled by calcitonin gene‐related peptide (CGRP, a marker for small DRG peptidergic neurons; Figure 1f), 48% by the purinergic receptor P2X3 (a marker for small non‐peptidergic neurons; Figure 1f), and 38% by neurofilament‐200 (NF200, a marker for medium/large cells and myelinated A*β*‐fibers; Figure 1f). Consistently, a cross‐sectional area analysis of neuronal somata revealed that approximately 50% of FTO‐positive neurons are small (<600 µm^2^ in area), 24% are medium (600–1200 µm^2^ in area), and 26% are large (>1200 µm^2^ in area) (Figure 1g). As expected, the number of FTO‐positive neurons in the ipsilateral L5 DRG on day 7 after SNL increased by 1.29‐fold compared with the corresponding sham rats (Figure 1h). On day 7 post‐SNL, all FTO‐positive neurons were positive for activating transcription factor 3 (ATF3), an injury marker ^[^ ^33^ ^]^ (Figure 1i). Collectively, these findings indicate that *Fto* gene is transcriptionally activated exclusively in the injured neurons of DRG after peripheral nerve injury.

### 2.2 Runx1 Promotes DRG *Fto* Gene Transcriptional Activity after SNL

**Figure 2:**
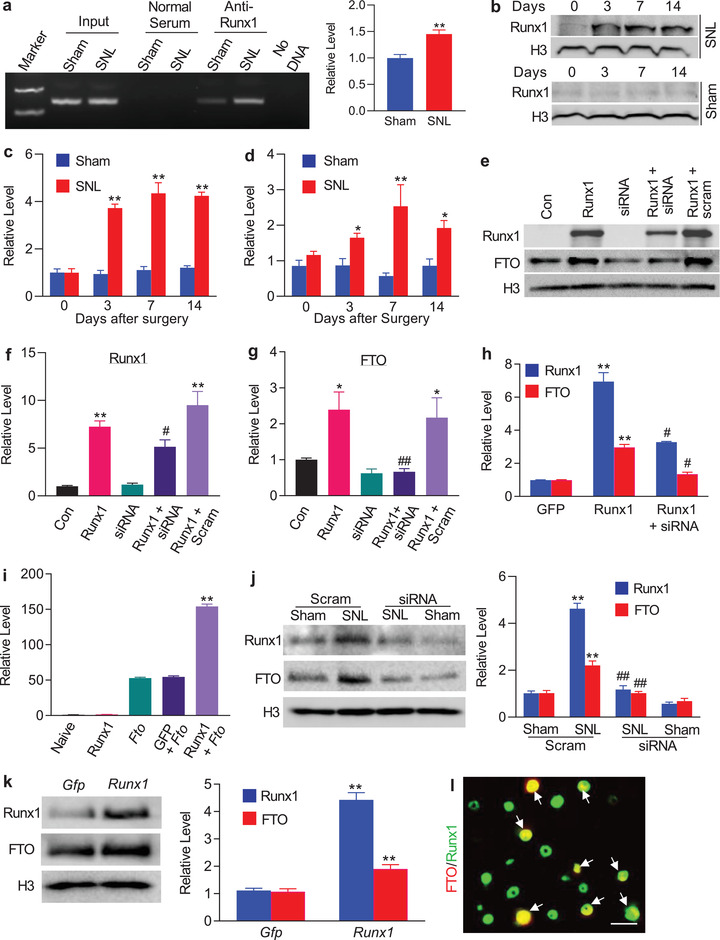
Runx1‐triggered *Fto* gene transcription in rat injured DRG following peripheral nerve injury. a) The *Fto* promotor fragment immunoprecipitated by rabbit anti‐Runx1 antibody in the ipsilateral L5 DRG on day 7 after SNL or sham surgery. Input: total purified fragments; *n* = 3 biological repeats (9 rats)/group. **p < 0.01 versus the sham group by two‐tailed unpaired Student's *t* test. b,c) Runx1 protein expression in the ipsilateral L5 DRG after SNL or sham surgery; *n* = 6 rats/time point/group. **p < 0.01 versus the corresponding control group (0 day) by two‐way ANOVA with repeated measures followed by post hoc Tukey test. d) Runx1 mRNA expression in the ipsilateral L5 DRG after SNL or sham surgery; *n* = 6 rats/time point/group. *p < 0.05, **p < 0.01 versus the corresponding control group (0 day) by two‐way ANOVA with repeated measures followed by post hoc Tukey test. e–g) Levels of Runx1 (e,f) and FTO (e,g) proteins in PC12 cells transfected with the vectors as shown. Con: control vector. *Runx1*: Runx1‐overexpressing vector. siRNA: *Runx1* siRNA. scram: scrambled siRNA. *n* = 5 biological repeats/treatment. *p < 0.05, **p < 0.01 versus control vector‐treated group. #*p* < 0.05 versus the *Runx1‐*overexpressing vector‐treated group. One‐way ANOVA with repeated measures followed by post hoc Tukey test. h) Levels of *Runx1* mRNA and *Fto* mRNA in rat lumbar DRG cultured neurons transduced with AAV5‐*Gfp* (*Gfp*), AAV5‐*Runx1* (*Runx1*), or AAV5‐*Runx1* plus *Runx1* siRNA (siRNA); *n* = 3 biological repeats/treatment. **p < 0.01 versus the corresponding AAV5‐*Gfp‐*treated group. #*p* < 0.05 versus the corresponding AAV5‐*Runx1‐*treated group. One‐way ANOVA with repeated measures followed by *post hoc* Tukey test. i) *Fto* gene promoter activities in PC12 cells transfected as shown. *Runx1*: Runx1‐overexpressing vector. *Fto*: vector expressing *Fto* gene promoter. *Gfp*: vector expressing *Gfp; n* = 3 biological repeats/treatment. **p < 0.01 versus the *Fto* plus *Gfp‐*treated group by one‐way ANOVA with repeated measures followed by post hoc Tukey test. j) Levels of Runx1 and FTO proteins in the ipsilateral L5 DRG on day 5 after SNL or sham surgery in the rats pre‐microinjected with *Runx1* siRNA (siRNA) or scrambled siRNA (Scram); *n* = 3 biological repeats (9 rats)/group. **p < 0.01 versus the corresponding scrambled siRNA‐treated sham group. ##*p* < 0.01 versus the corresponding scrambled siRNA‐treated SNL group. One‐way ANOVA with repeated measures followed by *post hoc* Tukey test. k) Levels of Runx1 and FTO proteins in the injected DRG 5 weeks after DRG microinjection of AAV5‐*Gfp* (*Gfp*) or AAV5‐*Runx1* (*Runx1*); *n* = 3 biological repeats (6 rats)/group. **p < 0.01 versus the AAV5‐*Gfp* group by two‐tailed unpaired Student's *t* test. l) Co‐expression of Runx1 (green) with FTO (red) in rat L5 DRG neurons (arrows); *n* = 3 rats. Scale bar: 40 µm.

Next, we examined how DRG *Fto* transcription was activated after peripheral nerve injury. We used online software TFSEARCH and identified a consensus runt‐related transcription factor 1 (Runx1)‐binding motif (_−856_TGTGGTTT_−849_) in the *Fto* promoter region. A chromatin immunoprecipitation (ChIP) assay showed that a *Fto* promoter fragment including the above binding motif could be amplified from the complex immunoprecipitated with Runx1 antibody in nuclear fractions from the DRG of sham rats (**Figure** **2**a). This amplification did not occur with control normal serum (Figure 2a) or after pre‐absorption of Runx1 antibody (data not shown), suggesting specific binding of Runx1 to the *Fto* promoter in DRG. SNL dramatically elevated the binding activity, as demonstrated by a 1.5‐fold increase in band density in the ipsilateral L5 DRG from the SNL rats compared to that from the sham rats on day 7 (Figure 2a). The increased binding activity may be attributed to time‐dependent increases in Runx1 expression at both mRNA and protein levels in the ipsilateral L5 DRG after SNL (Figure 2b–d). As expected, sham surgery did not alter the basal expression of Runx1 in the ipsilateral L5 DRG (Figure 2b,c). Neither SNL nor sham surgery changed basal binding activity or Runx1 expression in the contralateral L5 DRG and ipsilateral L4 DRG (data not shown).

To further demonstrate Runx1 regulation of *Fto* transcription, we first overexpressed full‐length *Runx1* in in vitro cultured PC12 cells, which express endogenous FTO. Runx1 overexpression significantly increased FTO protein expression (Figure 2e–g). This increase was abolished in the cells co‐transfected with full‐length *Runx1* vector and *Runx1*‐specific small interfering RNA (siRNA), but not in control scrambled siRNA (Figure 2e–g), indicating that FTO increase was a specific response to Runx1. We further confirmed the Runx1‐triggered increase of FTO in cultured DRG neurons that were transduced with recombinant adeno‐associated virus 5 (AAV5) expressing full‐length Runx1 (AAV5‐*Runx1*) (Figure 2h). The luciferase assay on transfected PC12 cells showed that co‐transfection of full‐length *Runx1* vector, but not control *Gfp* vector, markedly increased the activity of the *Fto* gene promoter (Figure 2i). Furthermore, blocking the SNL‐induced increase in DRG Runx1 through DRG pre‐microinjection of *Runx1* siRNA (but not control scrambled siRNA) not only attenuated the development of SNL‐induced mechanical allodynia, thermal hyperalgesia, and cold allodynia (Figure S2a–e, Supporting Information) but also reduced the SNL‐induced increase in FTO in the ipsilateral L5 DRG (Figure 2j). In addition, DRG overexpression of Runx1 through DRG microinjection of AAV5*‐Runx1* (but not control AAV5‐*Gfp*) increased the level of FTO in the injected DRGs (Figure 2k) in addition to the enhanced responses to mechanical, thermal, and cold stimuli (Figure S3a–c, Supporting Information) after viral microinjection in the rats without nerve injury. Given that all FTO‐labeled neurons were exclusively positive for Runx1 in rat DRG (Figure 2l), our findings strongly support that Runx1 participates in the nerve injury‐induced increase of DRG FTO.

### 2.3 Blocking Increased FTO in the Injured DRG Mitigates Neuropathic Pain

**Figure 3:**
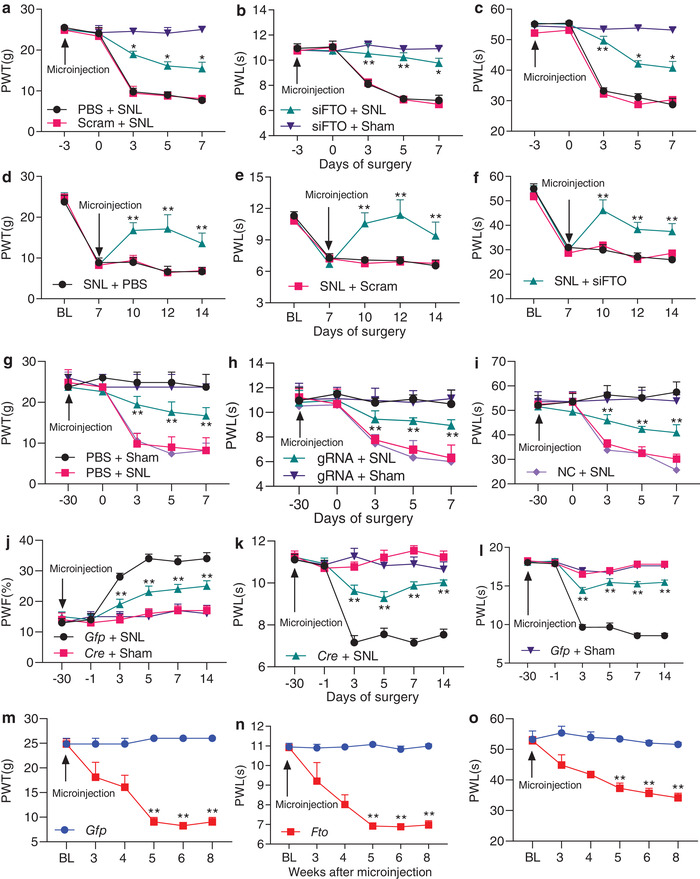
DRG increased FTO is required for development and maintenance of neuropathic pain. PWT: paw withdrawal threshold; PWL: paw withdrawal latency; PWF: paw withdrawal frequency. a−c) Effect of pre‐microinjection of *Fto* siRNA, scrambled siRNA (Scram) or PBS into the ipsilateral L5 DRG of the rats on the development of SNL‐induced mechanical allodynia (a), heat hyperalgesia (b), and cold allodynia (c) on the ipsilateral side; *n* = 6 rats/group. *p < 0.05, **p < 0.01 versus the PBS plus SNL group at the corresponding time points by two‐way ANOVA with repeated measures followed by post hoc Tukey test. d–f) Effect of post‐microinjection of *Fto* siRNA (siFTO), scrambled siRNA (Scram), or PBS into ipsilateral L5 DRG of the rats on the maintenance of SNL‐induced mechanical allodynia (d), heat hyperalgesia (e), and cold allodynia (f) on the ipsilateral side; *n* = 5 rats/group. **p < 0.01 versus the PBS plus SNL group at the corresponding time points by two‐way ANOVA with repeated measures followed by post hoc Tukey test. g–i) Effect of pre‐microinjection of AAV5‐*Fto* guide RNA (gRNA), AAV5‐negative control sequence (NC), or PBS into ipsilateral L5 DRG of rats on the development of SNL‐induced mechanical allodynia (g), heat hyperalgesia (h), and cold allodynia (i) on the ipsilateral side; *n* = 5 rats/group. **p < 0.01 versus the PBS plus SNL group at the corresponding time points by two‐way ANOVA with repeated measures followed by *post hoc* Tukey test. j–l) Effect of pre‐microinjection of AAV5‐*Cre* (*Cre*) or AAV5‐*Gfp* (*Gfp*) into the ipsilateral L4 DRG of *Fto^fl/fl^* mice on the development of SNL‐induced mechanical allodynia (j), heat hyperalgesia (k), and cold allodynia (l) on the ipsilateral side; *n* = 10 mice/group. *p < 0.01 versus the AAV5‐*Gfp*‐treated SNL mice at the corresponding time points by two‐way ANOVA with repeated measures followed by post hoc Tukey test. m–o) Ipsilateral paw withdrawal responses to mechanical (m), heat (n), and cold (o) stimuli at time points as shown after microinjection of AAV5‐*Fto* or AAV5‐*Gfp* into unilateral L4/5 DRGs in naive rats. *n* = 5 rats/group. *p < 0.01 versus the AAV5‐*Gfp*‐treated rats at the corresponding time points by two‐way ANOVA with repeated measures followed by post hoc Tukey test.

Does the increased FTO in the injured DRG participate in nerve injury‐induced pain hypersensitivities? To this end, we first examined the effect of blocking DRG FTO increase on the induction of SNL‐induced pain hypersensitivity. *Fto* siRNA and its control scrambled siRNA were microinjected into unilateral L5 DRG 3 days before SNL or sham surgery. Specificity and selectivity of *Fto* siRNA were demonstrated by its ability to knock down FTO, but not DNMT1, DNMT3a, and Kv1.4, in the ipsilateral L5 DRG on day 7 post‐surgery (Figure S4a, Supporting Information). Microinjection of *Fto* siRNA, but not scrambled siRNA, ameliorated SNL‐induced mechanical allodynia as indicated by an increase in paw withdrawal threshold to mechanical stimulation and impaired SNL‐induced heat hyperalgesia and cold allodynia as demonstrated by the increases in paw withdrawal latencies to heat and cold stimuli, respectively, on the ipsilateral side from day 3 to 7 post‐SNL compared to the vehicle (PBS)‐treated SNL rats (**Figure** **3**a–c). No changes were observed in basal mechanical, heat, or cold responses on the contralateral side of SNL rats and on both sides of sham rats following DRG microinjection of either siRNA (Figure 3a–c; Figure S4b,c, Supporting Information). We also observed the role of DRG FTO in the maintenance of neuropathic pain through microinjection of siRNA into unilateral L5 DRG on day 7 post‐surgery. Consistently, blunted mechanical allodynia, heat hyperalgesia, and cold allodynia were seen on days 10, 12, and 14 after SNL on the ipsilateral side of the *Fto* siRNA‐treated rats, but not of the scrambled siRNA‐treated rats (Figure 3d–f). Neither siRNA affected basal responses on the contralateral side (Figure 3d–f; Figure S4d,e, Supporting Information) and locomotor functions (Table S1, Supporting Information).

We also carried out a CRISPR/Cas9 system and designed the guide RNA (gRNA) that specifically and selectively knocked down *Fto* mRNA and FTO protein in in vitro PC12 cells and cultured rat DRG neurons (Figure S5a–d, Supporting Information). AAV5 that expressed the FTO‐specific gRNA (AAV5‐gRNA) or negative control sequence (AAV5‐NC) was microinjected into unilateral L5 DRG 30 days before SNL or sham surgery. Microinjection of AAV5‐gRNA, but not AAV5‐NC, significantly blocked SNL‐induced increase in the level of FTO on day 7 post‐SNL, although it did not affect basal level of FTO on day 7 post‐sham surgery, in the injured DRG (Figure S5e, Supporting Information). Like the *Fto* siRNA, DRG microinjection of AAV5‐gRNA impaired mechanical allodynia, heat hyperalgesia, and cold allodynia on the ipsilateral side from day 3 to 7 post‐SNL (Figure 3g–i). Consistently, this microinjection reduced the SNL‐induced dorsal horn central sensitization as indicated by abolishing the SNL‐induced increases in the phosphorylation of extracellular signal‐regulated kinase ½ (a marker for neuronal hyper‐activation ^[^ ^34^ ^]^) and glial fibrillary acidic protein (a marker of astrocyte activity ^[^ ^35^ ^]^) in the ipsilateral L5 dorsal horn (Figure S5f, Supporting Information). As expected, neither viral microinjections changed basal responses on the contralateral side of SNL rats and on both sides of sham rats (Figure 3g–i; Figure S5g,h, Supporting Information) and locomotor functions (Table S1, Supporting Information). Similar behavioral responses were seen after DRG viral microinjection in the CCI model (Figure S5i–l, Supporting Information).

Given that siRNA/gRNA may have potential off‐target effects, we further confirmed the role of DRG FTO in neuropathic pain using microinjection of AAV5‐*Cre* into the ipsilateral L4 DRG of *Fto* ^fl/fl^ mice 30 days before unilateral L4 SNL or sham surgery.^[^ ^36^ ^]^ AAV5‐*Gfp* was used as a control. The SNL‐induced increase in the level of FTO protein was blocked in the injured DRG of the *Fto* ^fl/fl^ mice injected with AAV5‐*Cre* 14 days after SNL (Figure S6a,b, Supporting Information). Although AAV5‐*Cre* injection reduced the basal level of FTO protein in the sham mice (Figure S6b, Supporting Information), basal paw withdrawal responses to mechanical, heat, and cold stimuli were similar between the two virus‐injected mice (Figure 3j–l). SNL‐induced mechanical allodynia, heat hyperalgesia, and cold allodynia were alleviated on the ipsilateral side of the AAV5‐*Cre*‐injected mice from days 3 to 14 post‐SNL (Figure 3j–l). DRG microinjection may produce cell damage, although the injected DRG retained their structural integrity and exhibited no changes in number of cells.^[^ ^37^, ^38^ ^]^ To exclude the possibility that the behavioral effects observed above were caused by tissue damage, we examined SNL‐induced pain behaviors in the conditional *Fto* knockout (KO) mice (Figure S7a, Supporting Information). As expected, SNL‐induced increase of FTO was absent in the injured DRG of *Fto* KO mice (Figure S7b, Supporting Information). Similar to AAV5‐*Cre*‐injected mice, *Fto* KO mice displayed the decreased paw withdrawal frequency to mechanical stimulation and increased paw withdrawal latency to heat or cold stimulation compared to *Fto* ^fl/fl^ mice on the ipsilateral side from days 3 to 14 post‐SNL (Figure S7c–f, Supporting Information). Basal responses on the contralateral side of SNL mice and on both sides of sham mice were not altered in the virus‐injected mice or genetic KO mice (Figure 3j–l; Figures S6c,d and S7c–I, Supporting Information). Both mice displayed normal locomotor activity (Table S1, Supporting Information).

Collectively, our data strongly suggest that increased FTO in the injured DRG is required for neuropathic pain development and maintenance.

### 2.4 Mimicking the Nerve Injury‐Induced DRG FTO Increase Produces Pain Hypersensitivity

We then asked whether the increased FTO in the injured DRG was sufficient for nerve injury‐induced pain hypersensitivity. To this end, we microinjected AAV5 that expressed full‐length *Fto* (AAV5‐*Fto*) into unilateral L4/5 DRGs of naïve adult rats. AAV5‐*Gfp* was used as a control. As expected, a marked increase in the level of FTO protein was detected 3 days after transfection of AAV5‐*Fto* in the cultured DRG neurons or 8 weeks post‐microinjection of AAV5‐*Fto* compared to the AAV5‐*Gfp*‐treated groups (Figure 8a,b, Supporting Information). Microinjection of AAV5‐*Fto* (not AAV5‐*Gfp*) produced mechanical allodynia, heat hyperalgesia, and cold allodynia as indicated by ipsilateral decreases in paw withdrawal thresholds in response to mechanical stimulation and in paw withdrawal latencies in response to heat and cold stimuli (Figure 3m–o). These decreases occurred 5 weeks post‐injection and persisted for at least 8 weeks (Figure 3m–o). Neither viral injections altered the basal paw withdrawal responses on the contralateral side (Figure S8c,d, Supporting Information) and affected locomotor functions (Table S1, Supporting Information). Our findings indicate that, in the absence of nerve injury, DRG FTO overexpression leads to neuropathic pain‐like symptoms.

### 2.5 Increased FTO Is Responsible for a Loss of m^6^A Sites in *Ehmt2* mRNA in the Injured DRG

**Figure 4:**
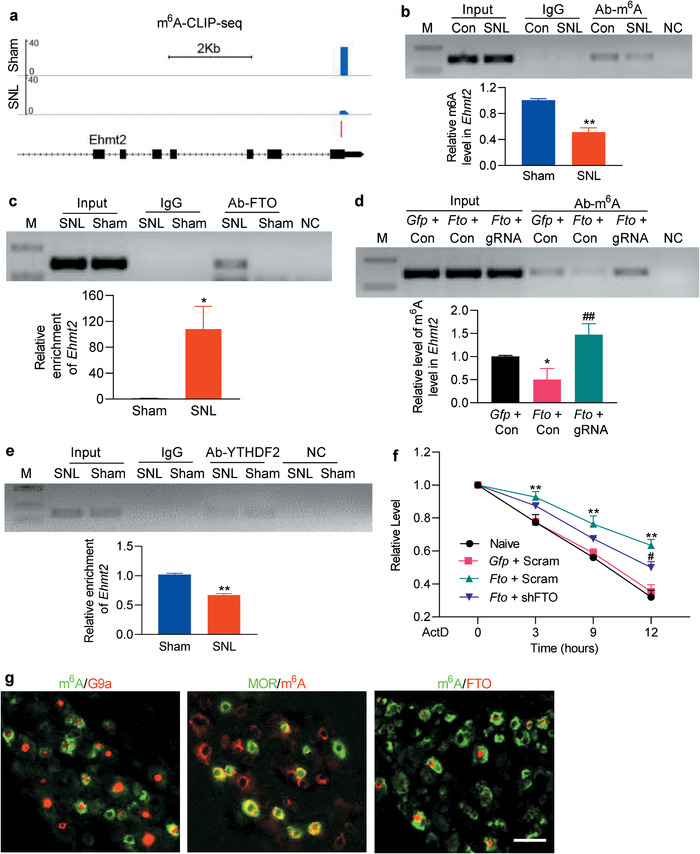
Increased FTO is responsible for a loss of m^6^A sites in *Ehmt2* mRNA in the injured DRG after SNL. M: ladder marker. NC: negative control (H_2_O). Input: total purified *Ehmt2* mRNA fragments from the ipsilateral L5 DRG or cultured DRG neurons. a) m^6^A‐eCLIP‐seq assay showed the example tracks of *Ehmt2* mRNA from the ipsilateral L5 DRGs on day 7 post‐SNL or sham surgery. m^6^A sites (blue) are indicated at the beginning of the 3′‐UTR near the stop codons. *n* = 3 biological repeats (20 rats/repeat)/group. b,c) Level of *Ehmt2* mRNA fragments containing at the beginning of the 3′‐UTR near the stop codons immunoprecipitated by rabbit anti‐m^6^A (b) or rabbit anti‐FTO (c), but not normal rabbit serum (IgG), in the ipsilateral L5 DRG on day 7 post‐SNL or sham surgery. Input: total purified fragment; *n* = 3 biological repeats (18 rats/repeat)/group. *p < 0.05, **p < 0.01 versus the corresponding sham group by two‐tailed unpaired Student's *t*‐test. d) Level of m^6^A in the *Ehmt2* mRNA fragment immunoprecipitated by anti‐m^6^A from rat cultured DRG neurons transduced by the viruses as shown. *Gfp*: AAV5‐*Gfp*; *Fto*: AAV5‐*Fto;* gRNA: AAV5‐*Fto* guide RNA; Con: AAV5‐control negative sequence; *n* = 3 biological repeats/group. One‐way ANOVA with repeated measures followed by post hoc Tukey test. *p < 0.05 versus the *Gfp* plus Con‐treated group. ##*p* < 0.01 versus the *Fto* plus Con‐treated group. e) Level of *Ehmt2* mRNA fragments immunoprecipitated by rabbit anti‐YTHDF2, but not normal rabbit serum (IgG), in the ipsilateral L5 DRG on day 7 post‐SNL or sham surgery; *n* = 3 biological repeats (18 rats/repeat)/group. **p < 0.01 versus the sham group by two‐tailed unpaired Student's *t*‐test. f) PC12 cells transfected by the vectors as shown were treated with actinomycin D (ActD; 5 µg mL^−1^) for the indicated times. The expression of *Ehmt2* mRNA was analyzed by real‐time PCR. *Gfp*: vector expressing *Gfp; Fto*: vector expressing full‐length *Fto*; Scram: vector expressing scrambled shRNA; shRNA: vector expressing *Fto* shRNA; *n* = 3 biological repeats. Two‐way ANOVA with repeated measures followed by post hoc Tukey test. **p < 0.01 versus naive group at the corresponding time points. #*p* < 0.05 versus the *Fto* plus Scram‐treated group at the corresponding time point. g) m^6^A‐labeled neurons are positive for G9a, mu opioid receptor (MOR), and FTO in the rat DRG *n* = 3 rats. Scale bar: 50 µm.

Next, we explored how increased DRG FTO contributed to neuropathic pain. Given that FTO functions as an “eraser” to remove m^6^A on RNAs,^[^ ^15^, ^16^, ^18^ ^]^ we carried out m^6^A‐enhanced cross‐linking and immunoprecipitation‐sequencing (m^6^A‐eCLIP‐seq) assay^[^ ^39^, ^40^ ^]^ to observe the changes of m^6^A sites across the transcriptome in the injured DRG on day 7 post‐SNL. Consistent with previous studies from cell lines,^[^ ^39^, ^40^ ^]^ m^6^A sites were identified across the 5′‐UTR, coding regions, 3′‐UTR, and introns under both sham and SNL conditions (Figure S9a, Supporting Information). After SNL, approximately 56% (2415/4325) transcripts displayed a loss of m^6^A sites, 21% (922/4325) transcripts displayed a gain of m^6^A sites, and 22% (953/4325) transcripts displayed both a gain and loss of m^6^A sites compared to the sham groups (Figure S9a, Supporting Information). About 1% (35/4325) transcripts did not show any changes in m^6^A sites between SNL and sham groups (data not shown). GO analysis revealed that these transcripts with increased or decreased m^6^A sites were enriched for regulation of catalytic activity and binding (Figure 9b,c, Supporting Information). *Ehmt2* mRNA that encodes the histone methyltransferase G9a exhibited a larger loss of m^6^A sites at the beginning of the 3′‐UTR near the stop codons from the injured DRG on day 7 after SNL compared to the sham group (**Figure** **4**a). This loss was further confirmed by the RNA immunoprecipitation (RIP) assay. The m^6^A enrichment at the beginning of the 3′‐UTR near the stop codons from the *Ehmt2* mRNA fragment was detected by the immunoprecipitation with anti‐m^6^A (but not normal serum) in the sham DRG (Figure 4b). However, the immunoprecipitative activity from the injured DRG on day 7 post‐SNL was significantly decreased (Figure 4b), indicating a loss of m^6^A sites in this fragment after SNL.

We then examined whether this loss was caused by the increased FTO in DRG neurons. The RIP assay revealed that basal binding between the *Ehmt2* mRNA fragment and FTO was rather weak in sham DRG (Figure 4c), but there was a striking elevation in the binding activity in the injured DRG on day 7 post‐SNL (Figure 4c). We further mimicked the SNL‐induced DRG FTO increase through transducing AAV5‐*Fto* into cultured DRG neurons and found that FTO overexpression produced a loss of m^6^A sites in the *Ehmt2* mRNA fragment, as evidenced by 50% decrease in immunoprecipitative activity between this fragment and anti‐m^6^A antibody compared to the control groups (Figure 4d; Figure S9d, Supporting Information). This decrease was reversed completely by blocking FTO overexpression in the cultured neurons co‐transduced with AAV5‐gRNA (Figure 4d; Figure S9d, Supporting Information). These findings suggest that the SNL‐induced loss of m^6^A in *Ehmt2* mRNA is attributed to the SNL‐induced increase in FTO in the injured DRG. YTHDF2 binds to m^6^A sites and is known to rapidly degrade m^6^A‐containing RNAs.^[^ ^24^, ^41^ ^]^ As expected, the binding activity between YTHDF2 and the *Ehmt2* mRNA fragment from the injured DRG on day 7 post‐SNL was significantly reduced by 33% compared to the sham group (Figure 4e). This reduction caused by increased FTO‐induced loss of m^6^A sites may promote the stabilization of *Ehmt2* mRNA. Indeed, when the transcription was halted with actinomycin D, the decay rate of *Ehmt2* mRNA was much slower and the level of G9a was markedly increased in the FTO‐overexpressing cells compared to that in control cells (Figure 4f; Figure S9e,f, Supporting Information). These effects could be attenuated by blocking FTO overexpression in the cells co‐transfected with vector expressing *Fto* shRNA (Figure 4f; Figure S9e,f, Supporting Information). Given that m^6^A‐labeled DRG neurons were positive for FTO, G9a, and mu opioid receptor (MOR; one of G9a‐controlled downstream targets^[^ ^12^, ^14^ ^]^) in the individual DRG neurons (Figure 4g), our data indicate that the increased FTO may be responsible for a loss of m^6^A sites in *Ehmt2* mRNA and then stabilize G9a upregulation in the injured DRG under neuropathic pain conditions.

### 2.6 Increased FTO Stabilizes the SNL‐Induced G9a Upregulation in the Injured DRG

**Figure 5:**
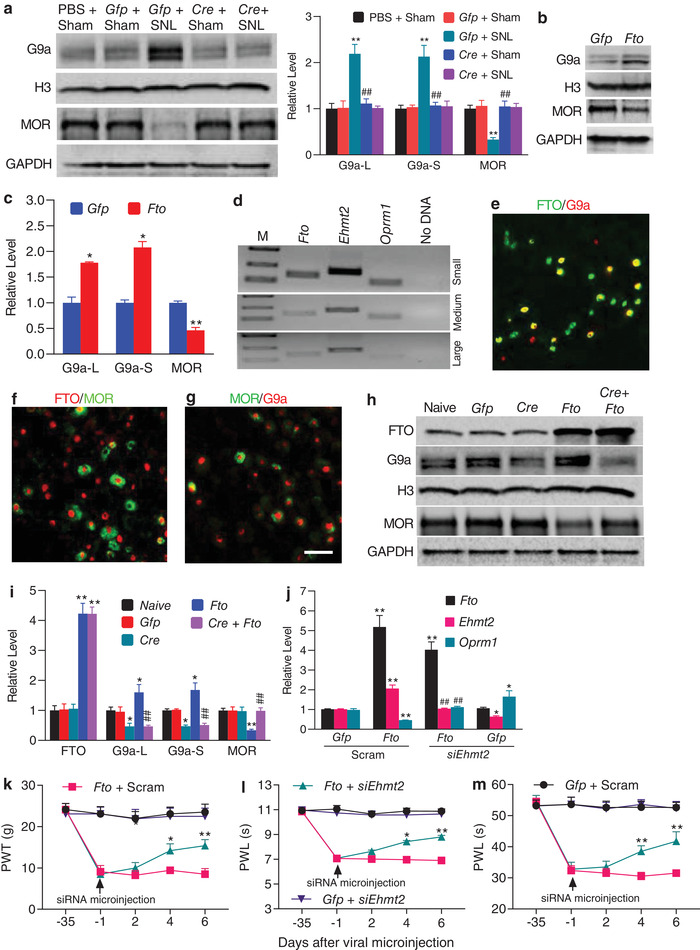
Increased FTO stabilizes SNL‐induced G9a upregulation in the injured DRG after SNL. a) Effect of pre‐microinjection of AAV5‐*Cre* (*Cre*) or AAV5‐*Gfp* (*Gfp*) into the ipsilateral L4 DRG of *Fto^fl/fl^* mice on SNL‐induced G9a upregulation and MOR downregulation in the ipsilateral L4 DRG on 14 post‐SNL or sham surgery. *n* = 3 biological repeats (12 mice)/group. One‐way ANOVA with repeated measures followed by post hoc Tukey test. **p < 0.01 versus the corresponding PBS‐treated sham group. ##*p* < 0.01 versus the corresponding AAV5‐*Gfp*‐treated SNL group. b,c) Effect of microinjection of AAV5‐*Fto* (*Fto*) or AAV5‐*Gfp* (*Gfp*) on the levels of G9a and MOR in the ipsilateral L4/5 DRGs of rats 8 weeks after microinjection; *n* = 3 biological repeats (6 rats)/group. *p < 0.05, **p < 0.01 versus the AAV5‐*Gfp*‐treated rats by two‐tailed unpaired Student's *t*‐test. d) Co‐expression of *Fto* mRNA, *Ehmt2* mRNA, and *Oprm1* mRNA in individual small and medium neurons from rat lumbar DRG; *n* = 3 biological repeats. e–g) Co‐localization of FTO with G9a (e) or MOR (f) as well as of G9a with MOR (g) in rat L5 DRG neurons *n* = 3 rats. Scale bar: 50 µm. h,i) Levels of FTO, G9a, and MOR expression in the cultured DRG neurons from *Ehmt2* ^fl/fl^ mice transduced with the viruses as indicated. *Gfp*: AAV5‐*Gfp*; *Cre*: AAV5‐*Cre*; *Fto*: AAV5‐*Fto*; *n* = 3 biological repeats/group. One‐way ANOVA with repeated measures followed by post hoc Tukey test. *p < 0.05, **p < 0.01 versus the corresponding naive group. ##*p* < 0.01 versus the corresponding AAV5‐*Fto*‐treated group. j) Effect of microinjection of *Ehmt2* siRNA (*siEhmt2*) or scrambled siRNA (Scram) into ipsilateral L4/5 DRGs on the expression of *Fto*, *Ehmt2*, and *Oprm1* mRNAs on day 6 after siRNA microinjection in the injected DRGs of the rats pre‐microinjected with AAV5‐*Fto* (*Fto*) or AAV5‐*Gfp* (*Gfp*); *n* = 3 biological repeats (6 rats)/group. One‐way ANOVA with repeated measures followed by post hoc Tukey test. *p < 0.05, **p < 0.01 versus the corresponding *Gfp* plus Scram group. ##*p* < 0.01 versus the corresponding *Fto* plus Scram group. k–m) Effect of microinjection of *Ehmt2* siRNA (*siEhmt2*) or scrambled siRNA (Scram) into ipsilateral L4/5 DRGs on ipsilateral paw withdrawal responses to mechanical (k), heat (l), and cold (m) stimuli at time points as shown in the rats pre‐microinjected with AAV5‐*Fto* (*Fto*) or AAV5‐*Gfp* (*Gfp*) into unilateral rat L4/5 DRGs. PWT: paw withdrawal threshold; PWL: paw withdrawal latency; *n* = 10 rats/group. *p < 0.05, **p < 0.01 versus the *Fto* plus Scram group at the corresponding time points by two‐way ANOVA with repeated measures followed by post hoc Tukey test.

To determine whether FTO stabilized DRG G9a upregulation after peripheral nerve injury, we examined the effect of blocking increased DRG FTO on G9a expression in the injured DRG. Microinjection of AAV5‐*Cre*, but not control PBS or AAV5‐*Gfp*, abolished the SNL‐induced increases in the levels of G9a's two protein isoforms and rescued the expression of MOR protein in the injured DRG of *Fto^fl/fl^* mice on day 14 post‐SNL (**Figure** **5**a; Figure S6b, Supporting Information). Similar results were observed in the injured rat DRG microinjected with AAV5‐gRNA, but not PBS or AAV5‐NC, on day 7 post‐SNL (Figure S10a, Supporting Information). Consistently, DRG overexpression of FTO in the AAV5‐*Fto*‐microinjected rats increased the levels of *Ehmt2* mRNA and G9a's two protein isoforms and decreased the level of MOR protein in the injected DRG, compared to the AAV5‐*Gfp*‐microinjected rats (Figure 5b,c). We also observed similar phenomenon in cultured DRG neurons transduced with AAV5‐*Fto* (Figure S10b, Supporting Information). Our single‐cell RT‐PCR assay showed co‐expression of *Fto* mRNA, *Ehmt2* mRNA, and *Oprm1* mRNA in individual small and medium (but not large) DRG neurons (Figure 5d). Double‐labeled staining revealed that approximately 58.3% (239/410) FTO‐labeled DRG neurons were positive for G9a and 30.6% (218/713) for MOR and that about 64.8% (162/250) G9a‐labeled DRG neurons were positive for MOR (Figure 5e–g). To exclude the possibility that FTO directly regulates MOR expression in DRG neurons, we overexpressed FTO through transduction of AAV5‐*Fto* with or without AAV5‐*Cre* into the cultured DRG neurons from *Ehmt2^fl/fl^* mice. As expected, the levels of G9a's two protein isoforms were increased and the amount of MOR was correspondingly decreased in the neurons transduced with AAV5‐*Fto* alone (Figure 5h,i). In contrast, AAV5‐*Fto* could not result in a decrease of MOR expression in the deficiency of G9a in the cultured neurons co‐transduced with AAV5‐*Cre* from *Ehmt2^fl/fl^* mice (Figure 5h,i). Moreover, blocking the FTO overexpression‐induced increase in DRG *Ehmt2* mRNA through post‐microinjection of *Ehmt2* siRNA (but not control scrambled siRNA) into the ipsilateral L4/5 DRGs starting at day 35 after DRG microinjection of AAV5‐*Fto* not only rescued the FTO overexpression‐induced downregulation of *Oprm1* mRNA in the injected DRGs (Figure 5j), but also attenuated the enhanced ipsilateral paw withdrawal responses to mechanical, heat, and cold stimuli on days 4 and 6 after siRNA microinjection (Figure 5k–m). Basal behavioral responses on the contralateral side were not affected (Figure S11a,b, Supporting Information). These findings indicate that G9a is required for FTO regulation of MOR in the DRG neurons. Collectively, the evidence described above suggests that increased FTO stabilizes the nerve injury‐induced upregulation of *Ehmt2* mRNA/G9a expression and participates in G9a‐triggered MOR downregulation in the injured DRG.

### 2.7 Increased FTO in the DRG Augments MOR‐Controlled Primary Afferent Neurotransmitter Release

**Figure 6:**
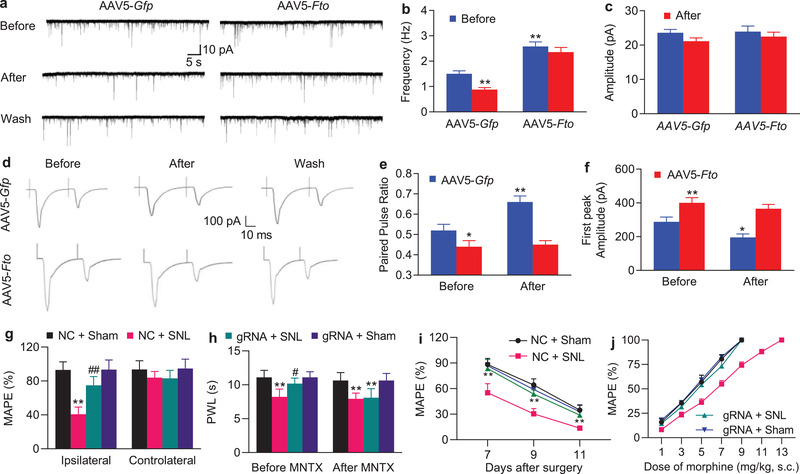
Increased FTO in the DRG augments neurotransmitter release from primary afferents and participates in the development of morphine analgesic tolerance in SNL rats. a) Example traces of miniature EPSC (mEPSC) recorded in lamina II dorsal horn neurons before and after DAMGO application and its wash out 5–6 weeks after microinjection of AAV5‐*Fto* or AAV5‐*Gfp* into unilateral L4/5 DRGs. b,c) Effects of DAMGO (1 µm) on the frequency (b) and amplitude (c) of mEPSC in lamina II neurons from the AAV5‐*Fto*‐treated group (*n* = 20 neurons, 14 rats) and AAV5‐*Gfp*‐treated group (*n* = 19 neurons, 13 rats). **p < 0.01 versus the AAV5‐*Gfp*‐treated group before DAMGO application by two‐way ANOVA with repeated measures followed by post hoc Tukey test. d). Example traces of C‐fiber input‐evoked EPSC in lamina II neurons before and after DAMGO application and its washout 5–6 weeks after microinjection of AAV5‐*Fto* or AAV5‐*Gfp* into unilateral L4/5 DRGs. e,f). Effects of DAMGO (1 µm) on the paired‐pulse ratio (e) and first peak amplitude of EPSC (f) in lamina II neurons from the AAV5‐*Fto*‐treated group (*n* = 16 neurons, 18 rats) and AAV5‐*Gfp*‐treated group (*n* = 16 neurons, 19 rats). *p < 0.05, **p < 0.01 versus the AAV5‐*Gfp*‐treated group before DAMGO application by two‐way ANOVA with repeated measures followed by post hoc Tukey test. g) Effect of pre‐microinjection of AAV5‐*Fto* guide RNA (gRNA) or AAV5‐negative control sequence (NC) into unilateral L5 DRG on morphine analgesia on the ipsilateral and contralateral sides 3 days post‐SNL or sham surgery. MAPE: maximal possible analgesic effect; *n* = 5 rats/group. Two‐way ANOVA with repeated measures followed by post hoc Tukey test. **p < 0.01 versus the AAV5‐NC‐treated sham group. ##*p* < 0.01 versus the AAV5‐NC‐treated SNL group. h) Effect of intraperitoneal injection of methylnaltrexone (MNTX) on the AAV5‐*Fto* guide RNA‐produced antinociception 3 days after SNL or sham surgery on the ipsilateral side. PWLs: paw withdrawal latencies; *n* = 5 rats/group. Two‐way ANOVA with repeated measures followed by post hoc Tukey test. **p < 0.01 versus the AAV5‐NC‐treated sham group. #*p* < 0.05 versus the AAV5‐NC‐treated SNL group. i) Morphine analgesic tolerance developed by subcutaneous injection of 10 mg kg^−1^ morphine twice daily for 4 days starting at day 7 post‐SNL or sham surgery on the ipsilateral side. MPAEs were measured 20 min after subcutaneous injection of 1.5 mg kg^−1^ morphine on the mornings of days 7, 9, and 11 post‐SNL or sham surgery; *n* = 5 rats/group. **p < 0.01 versus the AAV5‐NC‐treated SNL group at the corresponding time points by two‐way ANOVA with repeated measures followed by post hoc Tukey test. j) Dose‐response curve on the ipsilateral side on day 11 post‐SNL or sham surgery after subcutaneous injection of 10 mg kg^−1^ morphine twice daily for 4 days starting at day 7 post‐SNL or sham surgery from the AAV5‐NC‐ and AAV5‐gRNA‐injected rats; *n* = 5 rats/group.

G9a is a key player in neuropathic pain, at least in part due to its promotion of MOR‐controlled primary afferent neurotransmitter release.^[^ ^12^ ^]^ Thus, we tested whether mimicking the nerve injury‐induced DRG FTO increase augmented MOR‐controlled primary afferent neurotransmitter release. We measured the alternations in miniature excitatory postsynaptic current (mEPSC) frequencies, which reflected the changes in presynaptic neurotransmitter release,^[^ ^12^, ^42^ ^]^ recorded in lamina II neurons from ipsilateral L4/5 spinal cord slices of rats subjected to microinjection of AAV5‐*Fto* or control AAV5‐*Gfp* into the unilateral L4/5 DRGs 5–6 weeks post‐microinjection. mEPSC frequency in the AAV5‐*Fto*‐treated rats was markedly higher than that in the control AAV5‐*Gfp*‐treated rats (**Figure** **6**a,b). To verify whether this change was due to the decrease of MOR in primary afferents, we applied the selective MOR agonist DAMGO. DAMGO (1 µm) produced a great reduction in mEPSC frequency in the control rats (41.33%) than that in the AAV5‐*Fto*‐treated rats (8.53%) (Figure 6b). This effect was abolished after DAMGO washout (Figure 6a) or by pre‐addition of the selective MOR antagonist CTOP (data not shown). mEPSC amplitude was not changed in the two viral treated rats with or without DAMGO application (Figure 6c). We also recorded the EPSCs evoked by stimulation of the dorsal root (C‐fiber: 500 µA intensity and 1 Hz) with a paired‐pulsed protocol. The paired‐pulse ratio (PPR), which reflects the probability of primary afferent neurotransmitter release,^[^ ^12^, ^42^ ^]^ was defined by dividing the second peak amplitude by the first one. The PPR markedly declined in the AAV5‐*Fto*‐treated rats compared to the control rats (Figure 6d,e). Additionally, the first peak amplitude of the evoked EPSC in the AAV5‐*Fto*‐treated rats was higher than that in the control rats (Figure 6d,f). DAMGO application significantly increased the PPR by 26.16% and decreased the first peak amplitude by 32.1% in the control rats, but only 2.4% and 8.65%, respectively, in the AAV5‐*Fto*‐treated rats (Figure 6d–f). After DAMGO washout, its effect was abolished (Figure 6d) or by pre‐application with CTOP (data not shown). The evidence indicates that increased FTO in the DRG augments neurotransmitter release from primary afferent terminals likely due to G9a‐triggered MOR downregulation in the primary afferents.

### 2.8 Blocking Increased DRG FTO Improves MOR‐Induced Analgesia and Tolerance under Neuropathic Pain Conditions

Neuropathic pain patients often exhibit limited effectiveness of MOR agonists and increased relevant side effects such as tolerance development.^[^ ^43^ ^]^ Given that blocking the SNL‐induced DRG FTO increase rescued MOR expression in the injured DRG (Figure 5a), we finally questioned whether this blockade improved morphine‐induced analgesia in the SNL rats. Consistent with previous studies,^[^ ^35^, ^44^ ^]^ the analgesic effect caused by subcutaneous (s.c.) injection of morphine (1.5 mg kg^−1^) on day 3 after SNL was markedly reduced compared to that in sham group on the ipsilateral side of the AAV5‐NC‐treated rats (Figure 6g). This reduction was significantly rescued on the ipsilateral side of the AAV5‐gRNA‐treated rats (Figure 6g). As expected, morphine led to robust analgesia on the ipsilateral side of the AAV5‐gRNA‐treated sham rats and on the contralateral side of all treated groups (Figure 6g). We also intraperitoneally injected methylnaltrexone bromide (MNTX, a peripheral MOR antagonist; 5 mg kg^−1^) on day 3 post‐SNL or sham surgery. The AAV5‐gRNA‐caused antinociceptive effect on SNL‐induced thermal hyperalgesia was absent 30 min after MNTX administration (Figure 6h). MNTX at the dose used did not change the SNL‐induced heat hyperalgesia in the AAV5‐NC‐treated SNL rats and basal responses on the ipsilateral side of the sham groups (Figure 6h) and on the contralateral side of all treated groups (data not shown). These data suggest the involvement of peripheral MOR in anti‐nociception caused by blocking DRG FTO increase under neuropathic pain conditions.

We further questioned whether blocking DRG FTO increase alleviated morphine‐induced tolerance after SNL. Repeated s.c. morphine injections twice daily (10 mg kg^−1^) for 4 days starting from day 7 after SNL produced a time‐dependent decrease in morphine analgesia from day 7 to 11 post‐injection in the AAV5‐NC‐treated rats (Figure 6i). However, this decrease was markedly attenuated in the AAV5‐gRNA‐treated rats (Figure 6i). This effect was also confirmed by a great leftward shift in the cumulative dose‐response curve of morphine analgesia in the AAV5‐gRNA‐treated SNL rats compared with the AAV5‐NC‐treated SNL rats on day 11 post‐injection (Figure 6j). In SNL rats, the ED_50_ value of morphine analgesia in the AAV5‐gRNA‐treated group (4.8 mg kg^−1^) was substantially lower than that in the AAV5‐NC‐treated group (6.8 mg kg^−1^) (Figure 6j). Together, our findings indicate that blocking DRG FTO increase improves morphine analgesia and its tolerance development under neuropathic pain conditions.

## 3 Discussion

In this study, we provide the first evidence to our knowledge that peripheral nerve injury leads to an increase in FTO expression through an activation of the transcription factor Runx1 in the injured DRG neurons. This increase correlates with a loss of m^6^A sites in *Ehmt2* mRNA and an elevation of G9a protein in the DRG and leads to neuropathic pain symptoms. Blocking this increase reverses a loss of m^6^A sites in *Ehmt2* mRNA and destabilizes the nerve injury‐induced G9a upregulation in the injured DRG and alleviates nerve injury‐associated pain hypersensitivities. DRG FTO likely contributes to the mechanisms of neuropathic pain through stabilizing G9a expression in the DRG.

FTO is expressed in the nervous system including DRG.^[^ ^45^ ^]^ A recent report showed that FTO was enriched in the nucleus, cytoplasm, and axons of the cultured DRG neurons from mouse embryonic day 13.^[^ ^46^ ^]^ An in vitro study of COS7 cells transfected with exogenous GFP‐FTO also revealed the localization of FTO in both nucleus and cytoplasm.^[^ ^47^ ^]^ Interestingly, other two in vitro studies reported that endogenous FTO was entirely localized in the nuclei of COS7 cells or HeLa cells.^[^ ^48^, ^49^ ^]^ The present study showed that FTO was localized predominantly in the nuclei of adult rat DRG neurons as it co‐expressed with DAPI in the *β*‐tubulin III‐labeled individual neurons and was not detected in GS‐positive satellite cells. Although FTO has the potential expression in DRG immune cells (e.g., NK cells and monocytes) and played an active role in nerve degeneration after nerve injury,^[^ ^50^, ^51^ ^]^ morphologic observation of the FTO‐labeled DRG cells does not support this expectation. Our conclusion is further confirmed by the evidence that FTO co‐localized with CGRP, P2X3, or NF200, all of which are distributed exclusively in DRG neurons. Our findings are consistent with those reported in previous studies that showed a neuronal nuclei location for FTO in adult central nervous system, including neocortex, hippocampus, and midbrain.^[^ ^27^, ^52^, ^53^ ^]^

**Figure 7:**
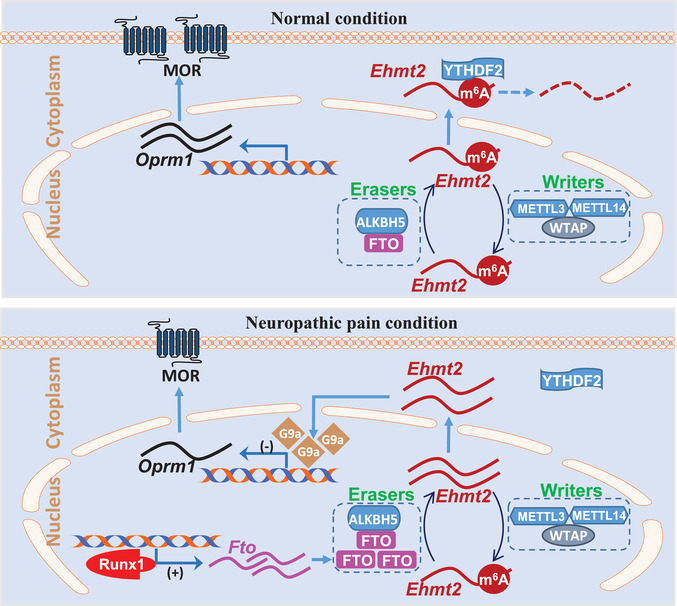
Proposed mechanism by which FTO participates in neuropathic pain. In normal dorsal root ganglion (DRG) neurons, FTO expression is relative low. The m^6^A level in *Ehmt2* RNA is maintained by both m^6^A writers and erasers. The m^6^A reader YTHDF2 degrades *Ehmt2* RNA, resulting in low expression of G9a and normal expression of mu opioid receptor (MOR). In contrast, FTO is up‐regulated through Runx1‐triggered *Fto* gene activation in the injured DRG after nerve injury. The increased FTO reduces basal level of m^6^A in *Ehmt2* RNA, losses the binding to YTHDF2, and stabilizes the increased expression of *Ehmt2* mRNA and G9a protein. The latter epigenetically inhibits *Oprm1* transcriptional activation and down‐regulates MOR in DRG neuronal cell membranes and primary afferent terminals, resulting in the enhanced DRG neuronal excitability and primary afferent neurotransmitter/modulator release.

The *Fto* gene is transcriptionally activated in the injured DRG after peripheral nerve injury. *Fto* mRNA and its protein increased in the injured DRG, but not in intact (uninjured) DRG or contralateral DRG, after SNL or CCI. Our findings further verified RNA sequencing results after SNL or spared nerve injury (SNI) reported previously.^[^ ^4^, ^54^ ^]^ Interestingly, unlikely in SNI DRG,^[^ ^54^ ^]^ there were no changes in the levels of *Mettl3*, *Mettl14*, *Alkbh5*, *Wtap*, and *Ythdf2* mRNAs ^[^ ^4^ ^]^ as well as their proteins in SNL DRG. It appears that these gene expression varies with distinct neuropathic pain models. Unexpectedly, CFA‐induced peripheral inflammation did not alter basal FTO expression in the DRG on either ipsilateral or contralateral side, although it is unknown if its enzymatic activity was changed after CFA injection. It appears that *Fto* gene activation is nerve injury‐specific. Moreover, the transcription factor Runx1, which was required for neuropathic pain genesis,^[^ ^55^ ^]^ triggered this activation after peripheral nerve injury (**Figure** **7**). Whether additional transcription factors (e.g., C/EBP*α* ^[^ ^56^ ^]^) are also involved in DRG *Fto* gene activation following nerve injury is unknown. In addition, other potential possibilities such as epigenetic modifications and/or increased mRNA stability that may lead to an increase of DRG *Fto* mRNA could not be ruled out under neuropathic pain conditions.

FTO is a demethylase especially for m^6^A sites in RNAs. Our eCLIP‐seq assay revealed that majority of transcripts lost m^6^A sites across the 5′‐UTR, coding regions, 3′‐UTR, and even introns in the injured DRG after SNL. These losses correlate with the SNL‐induced DRG FTO increase. Unexpectedly, some transcripts gained m^6^A sites in the injured DRG after SNL. How m^6^A sites were increased in these transcripts under the conditions of detectable increase of FTO expression without basal alterations of METTL3/14 and WTAP expression in the injured DRG after SNL remains unclear. Even more puzzling was how SNL simultaneously led to a gain and loss of m^6^A sites in some individual transcripts in the injured DRG. All of these questions merit further investigations.

The increased FTO in the injured DRG may contribute to the development and maintenance of neuropathic pain by stabilizing nerve injury‐induced G9a upregulation in the injured DRG (Figure 7). Although FTO may target many mRNAs, the present study focused on *Ehmt2* mRNA because *Ehmt2* mRNA and G9a play a key role in neuropathic pain genesis.^[^ ^3^, ^11^, ^12^, ^13^, ^14^ ^]^ Additionally, our m^6^A‐eCLIP‐seq assay showed a large loss of m^6^A in *Ehmt2* mRNA. We demonstrated that increased FTO erased m^6^A sites in *Ehmt2* mRNA, resulting in a loss in the binding of *Ehmt2* mRNA to YTHDF2 in the injured DRG (Figure 7). Given that YTHDF2 promoted the degradation of its binding RNAs,^[^ ^24^, ^41^ ^]^ the increased FTO likely stabilized nerve injury‐induced upregulation of G9a in the injured DRG (Figure 7). Indeed, FTO overexpression delayed the degradation of *Ehmt2* mRNA in in intro cell line. Blocking SNL‐induced DRG FTO increase abolished SNL‐induced upregulation of G9a and rescued MOR expression in the injured DRG. Mimicking this increase removed m^6^A sites in *Ehmt2* mRNA, increased the level of G9a, and reduced the amount of MOR in the DRG. Moreover, blocking the FTO overexpression‐induced increases in DRG *Ehmt2* mRNA and G9a not only rescued the FTO overexpression‐induced downregulation of DRG MOR but also attenuated the FTO overexpression‐induced pain hypersensitivities. Thus, the anti‐hyperalgesia caused by blocking DRG FTO increase is likely resulting from destabilizing nerve injury‐induced G9a upregulation in the injured DRG. It was unexpected that DRG microinjection of *Fto* siRNA or AAV5‐gRNA did not markedly affect basal expression of FTO in the sham rats, although they were effective in cultured DRG neurons. Why *Fto* siRNA or AAV5‐gRNA had no effect on basal FTO expression in vivo is unclear but could be due to lower level of DRG FTO expression under normal conditions. AAV5 or siRNA microinjection given at current volume (1 µL) and concentration/title likely cannot produce more basal reduction in DRG FTO. Without DRG G9a upregulation, no alterations would occur in DRG MOR expression, a MOR‐controlled increase in neurotransmitter release from primary afferents, and subsequent dorsal horn central sensitization.^[^ ^12^, ^13^, ^14^ ^]^ Consistent with this conclusion, blocking DRG FTO increase attenuated the SNL‐induced increase in markers for dorsal horn central sensitization, rescued a loss of morphine analgesia, and delayed the development of morphine analgesic tolerance in SNL rats. In addition, the anti‐hyperalgesic effect caused by blocking DRG FTO increase on SNL‐induced pain hypersensitivity was diminished by antagonizing the peripheral MOR. FTO did not directly regulate MOR as FTO overexpression failed to downregulate MOR expression in the absence of G9a expression in the DRG neurons. Together, our results suggest that DRG FTO participates in the mechanism of neuropathic pain at least in part through epigenetic silence of MOR caused by FTO‐mediated stabilization of nerve injury‐induced G9a upregulation in the injured DRG (Figure 7). However, other potential mechanisms of FTO participation in neuropathic pain cannot be ruled out. Our m^6^A‐eCLIP assay showed that, besides *Ehmt2* transcript, other transcripts also exhibited a loss and/or gain of m^6^A sites in the injured DRG after SNL. Whether these transcripts are targeted by FTO and involved in FTO function in neuropathic pain remains to be studied. Additionally, FTO may regulate gene expression via m^6^A DNA modification.^[^ ^58^ ^]^ Thus, DRG FTO contributes to neuropathic pain likely through its triggered multiple mechanisms.

In summary, our study demonstrated that blocking the nerve injury‐induced DRG FTO increase mitigated neuropathic pain without changing basal or acute pain and locomotor function. This blockage also improved morphine analgesia and alleviated morphine analgesic tolerance development under neuropathic pain conditions. Given that the FTO inhibitor meclofenamic acid was approved by FDA as a nonsteroidal anti‐inflammatory drug,^[^ ^59^, ^60^ ^]^ blocking DRG FTO may have a potential clinical application in neuropathic pain management.

## 4 Experimental Section

##### Animals

Male Sprague‐Dawley rats (weighing 200–250 g or 2–3 months old for in vivo experiments and weighing 60–100 g or 21–30 days old for in vitro culture) were purchased from Charles River Laboratories (Willington, MA). *Fto* ^fl/fl^ mice (provided by Dr. Rudy Leibei from Columbia University), *Ehmt2* ^fl/fl^ mice (provided by Dr. Eric J Nestler from Icahn School of Medicine at Mount Sinai), and Advillin ^Cre/+^ mice (provided by Dr. Fan Wang from Duke University) were bred in the facility. *Fto* ^fl/fl^ mice and *Ehmt2* ^fl/fl^ mice were fully backcrossed to C57BL/6 J mice and were homozygous for a floxed *Fto* and *Ehmt2* allele. Male sensory‐specific *Cre* line Advillin^Cre/+^ mice were crossed with female *Fto* ^fl/fl^ mice to obtain *Fto* conditional knockout (KO) mice. Male mice weighing 25–30 g (2–3 months old) were used for the experiments. The central housing facility at Rutgers New Jersey Medical School provided all animals for a standard 12 h light/dark cycle, with water and food pellets available ad libitum. All procedures were approved by the Animal Care and Use Committee at the Rutgers New Jersey Medical School and are also consistent with the ethical guidelines of the National Institutes of Health and the International Association for the Study of Pain. Every effort was made to minimize animal suffering and the number of animals used. All of the experimenters were blind to treatment condition.

##### Animal Models

For neuropathic pain models, L_5_ spinal nerve ligation (SNL) and sciatic nerve chronic constriction injury (CCI) in rats and L_4_ SNL in mice were carried out as published previously.^[^ ^36^, ^38^, ^61^, ^62^ ^]^ In brief, for the SNL model, after the animals were anesthetized with isoflurane, an incision on the lower back was made and the lumbar transverse process was removed. The underlying spinal nerve (L_4_ in mice and L_5_ in rats) was isolated and ligated with a 3‐0 silk thread in rats or 7‐0 silk thread in mice. The ligated nerve was then transected distal to the ligature. The skin and muscles were finally closed in layers. For the CCI model, unilateral exposed sciatic nerve was loosely ligated with 3‐0 silk thread at four sites with an interval of about 1 mm proximal to trifurcation of the sciatic nerve. The sham groups underwent identical procedures of the SNL or CCI group, but without the ligature/transection of the respective nerve. The complete Freund's adjuvant (CFA)‐induced chronic inflammatory pain model in rats was performed as described previously.^[^ ^32^, ^57^, ^63^ ^]^ A 100 µL solution of CFA (1 mg mL^−1^ *Mycobacterium tuberculosis*; Sigma, St. Louis, MO) was subcutaneously injected into the plantar side of unilateral hind paw.

##### Behavioral Tests

Mechanical, heat, and cold tests as well as locomotor function test were carried out as described.^[^ ^38^, ^61^, ^62^, ^64^ ^]^ There was a 1‐h interval between two tests.

Paw withdrawal thresholds (in rats) or frequencies (in mice) in response to mechanical stimuli (calibrated von Frey filaments) were first measured. In brief, the animals were placed in individual Plexiglas chambers on an elevated mesh screen. For rats, calibrated von Frey filaments (Stoelting Co., Wood Dale, IL) in log increments of force (0.69, 1.20, 2.04, 3.63, 5.50, 8.51, 15.14, and 26 g) were used to stimulate the plantar surface of the rats’ left and right hind paws. The 3.63 g stimulus was used first. If a negative response occurred, the next larger von Frey hair was applied; if a positive response was seen, the next smaller von Frey hair was applied. The application was terminated when i) a negative response was seen with the 26 g stimulation or ii) three stimuli were used after the first positive response. Based on the formula provided by Dixon,^[^ ^65^ ^]^ paw withdrawal threshold was calculated by converting the pattern of positive and negative responses to a 50% threshold value. For mice, two calibrated von Frey filaments (0.07 and 0.4 g) were used to stimulate the hind paw for approximately 1 s, and each stimulation was repeated ten times to both hind paws with 5 min interval. Paw withdrawal response in each of these ten applications was represented as a percent response frequency ([number of paw withdrawals/10 trials] × 100 = % response frequency), and this percentage was obtained as an indication of the amount of paw withdrawal.

Paw withdrawal latencies to noxious thermal stimulation were then examined with a Model 336 Analgesia Meter (IITC Inc. Life Science Instruments. Woodland Hills, CA). Briefly, the animal was placed in an individual Plexiglas chamber on a glass plate. A beam of light through a hole in the light box of Model 336 Analgesic Meter through the glass plate was used to stimulate the middle of the plantar surface of each hind paw. The light beam was automatically turned off when the animal withdrew its foot. The paw withdrawal latency was recorded by the length of time between the start of the light beam and the foot withdraw. Each test was repeated five times at 5 min intervals for the paw on each side. To avoid tissue damage to the hind paw, a cut‐off time of 20 s was applied.

Paw withdrawal latencies to noxious cold (0 °C) were examined when the animal was placed in an individual Plexiglas chamber on a cold aluminum plate, the temperature of which was monitored continuously by a thermometer. The paw withdrawal latency was recorded by the length of time between the placement of the hind paw on the plate and a flinching of the paw. Each test was repeated three times at 10 min intervals for the paw on the ipsilateral side. To avoid tissue damage, a cut‐off time of 60 s for rats or 20 s for mice was used.

Locomotor functions, including placing, grasping, and righting reflexes, were examined after above‐described behavioral tests. 1) Placing reflex: The placed positions of the hind limbs were slightly lower than those of the forelimbs, and the dorsal surfaces of the hind paws were brought into contact with the edge of a table. Whether the hind paws were placed on the table surface reflexively was recorded; 2) grasping reflex: after the animal was placed on a wire grid, whether the hind paws grasped the wire on contact was recorded; 3) righting reflex: when the animal was placed on its back on a flat surface, whether it immediately assumed the normal upright position was recorded. Each trial was repeated five times with 5 min interval and the scores for each reflex were recorded based on counts of each normal reflex.

##### Morphine‐Induced Analgesia and Tolerance

Morphine analgesia was established in rats as reported previously.^[^ ^42^, ^66^ ^]^ Briefly, the morphine (1.5 mg kg^−1^; WEST‐WARD, Eatontown, NJ) was injected subcutaneously (s.c.) 3 days after SNL or sham surgery. Thermal test as described above was carried out before surgery (baseline latency) and 30 min after morphine injection (response latency). The cut‐off time was 20 s to avoid tissue damage. The percentage of maximal possible analgesic effect (% MPAE) was calculated as follows: % MPAE = ([response latency‐baseline latency]/[cut‐off time‐baseline latency]) × 100%. Additionally, a peripheral MOR antagonist methylnaltrexone bromide (MNTX; 5 mg kg^−1^, dissolved in saline; Medchemexpress, Monmouth Junction, NJ) was administered intraperitoneally (i.p.) 3 days after SNL or sham surgery. Thermal tests were carried out before and 30 min after MNTX injection.

Analgesic tolerance was induced in rats as previously described.^[^ ^42^, ^67^, ^68^ ^]^ In brief, morphine (10 mg kg^−1^) was injected s.c. twice daily for 4 consecutive days starting on day 7 post‐SNL or sham surgery. Thermal test was performed before morphine injection and 20 min after s.c. injection of an inducing dose of morphine (1.5 mg kg^−1^ in SNL or sham rats) on the mornings of days 7, 9, and 11 post‐SNL or sham surgery. Cumulative morphine dose‐response test was carried out as described previously ^[^ ^42^, ^67^, ^68^ ^]^ on the afternoon of day 11 post‐SNL or sham surgery. Rats were injected s.c. with a low dose of morphine (1 mg kg^−1^), and analgesia was assessed 20 min later by the thermal test. Rats that were not analgesic at the first dose then received a second dose (cumulative dosing increase by 0.3 log units) and were tested 20 min afterward. This procedure was repeated until the rats failed to move their paw within the cutoff time or no additional increase in paw withdrawal latency was noted from one dose to the next.

##### DRG Microinjection

DRG microinjection was carried out in the same manner as described previously.^[^ ^38^, ^61^ ^]^ In brief, after the animal was anesthetized with isoflurane, a midline incision in the lower lumbar back region was made and the lumbar articular process was exposed and then removed. The exposed DRG was injected with viral solution (1–1.5 µL in rats and 0.5–1 µL in mice) through a glass micropipette connected to a Hamilton syringe. The pipette was retained for 10 min after injection. Animals showing signs of paresis or other abnormalities were excluded. The injected DRGs were stained with hematoxylin/eosin to examine the integrity of their structure and whether they contained visible leukocytes.

##### Cell Line Culture and Transfection

PC12 (ATCC) cells were cultured in Dulbecco's Modified Eagle's Medium (DMEM; Gibco/ThermoFisher Scientific, Waltham, MA) containing 5% horse serum and 5% v/v fetal bovine serum (FBS; Gibco/ThermoFisher Scientific) at 37 °C in a humidified incubator with 5% CO_2_. The plasmids, gRNAs and shRNAs were transfected into the PC12 cells with Lipofectamine 2000 (Invitrogen, Carlsbad, CA) according to the manufacturer's instructions and the previous work.^[^ ^38^ ^]^

##### DRG Neuronal Culture and Transduction

Primary DRG neuronal cultures from adult rats/mice and viral transfection were performed as described.^[^ ^38^ ^]^ Briefly, after the animals were euthanized with isoflurane, all DRGs were collected in cold Neurobasal Medium (Gibco/ThermoFisher Scientific) containing 10% fetal bovine serum (JR Scientific, Woodland, CA), 100 units mL^−1^ penicillin, and 100 µg mL^−1^ streptomycin (Quality Biological, Gaithersburg, MD). The DRGs were then treated with enzyme solution (5 mg mL^−1^ dispase, 1 mg mL^−1^ collagenase type I in Hanks’ balanced salt solution without Ca^2+^ and Mg^2+^ [Gibco/ThermoFisher Scientific]). After trituration and centrifugation, the dissociated neurons were resuspended in mixed Neurobasal Medium and plated in a six‐well plate coated with 50 µg mL^−1^ poly‐d‐lysine (Sigma). The cells were incubated at 95% O_2_, 5% CO_2_, and 37 °C. On the second day, 2–10 µL of virus (titer ≥ 1 × 10^12^ GC/mL) was added to each well. The neurons were collected 3 days later.

##### Reverse Transcription‐PCR

Quantitative real‐time reverse transcription (RT)‐PCR was performed as described.^[^ ^38^, ^67^ ^]^ To achieve enough RNA, four unilateral mouse DRGs or two unilateral rat DRGs were pooled together. Total RNA from the tissues or the cultured samples was purified using miRNeasy kit with on‐column digestion of genomic DNA (Qiagen, Valencia, CA) and reverse‐transcribed using the ThermoScript reverse transcriptase (Invitrogen/ThermoFisher Scientific), oligo (dT) primers, or specific RT‐primers (Table S2, Supporting Information). Template (1 µL) was amplified by real‐time PCR using the primers listed in Table S2, Supporting Information (Integrated DNA Technologies). Each sample was run in triplicate in a 20 µL reaction with 250 nm forward and reverse primers, 10 µL of SsoAdvanced Universal SYBR Green Supermix (Bio‐Rad Laboratories, Hercules, CA), and 20 ng of cDNA. Reactions were carried out in a BIO‐RAD CFX96 real‐time PCR system. *Gapdh* was used as an internal control for normalization. Ratios of mRNA levels from the treated groups or different time points to mRNA level from control/naive (0 day) group were calculated using the ΔCt method (2^−ΔΔCt^). All data were normalized to *Gapdh*, as it was demonstrated to be stable even after peripheral nerve injury insult.

Single‐cell real‐time RT‐PCR was carried out as described.^[^ ^38^, ^67^ ^]^ In brief, the freshly cultured rat DRG neurons were first prepared. Four hours after plating, under an inverted microscope fit with a micromanipulator and microinjector, a single living large (>35 µm), medium (25–35 µm), and small (<25 µm) DRG neuron was collected in a PCR tube with 9–10 µL of cell lysis buffer (Signosis, Sunnyvale, CA). After centrifugation, the supernatants were collected and divided into three PCR tubes for *Fto*, *Ehmt2*, and *Oprm1* genes, respectively. The remaining real‐time RT‐PCR procedure was performed based on the manufacturer's instructions with the single‐cell real‐time RT‐PCR assay kit (Signosis). All primers used are listed in Table S2, Supporting Information.

##### Plasmid Constructs and Virus Production

By using the SuperScript III One‐Step RT‐qPCR System with the Platinum *Taq* High Fidelity Kit (Invitrogen/ThermoFisher Scientific) and the primers (Table S2, Supporting Information), rat full‐length *Fto* cDNA or *Runx1* cDNA was synthesized and amplified from total RNA of rat DRG. A Rat *Fto* shRNA duplex corresponding to bases 588‐608 from the open reading frame of rat *Fto* mRNA (GenBank accession number NM_001039713) was designed (Table S2, Supporting Information). A mismatch shRNA with a scrambled sequence and no known homology to a rat gene (scrambled shRNA) was used as a control (Table S2, Supporting Information). shRNA oligonucleotides listed in Table S2, Supporting Information, were annealed and were inserted to pAAV‐shRNA‐Ctrl‐EYFP. A 21 nt long rat *Fto* guide RNA with PAM sequence (gRNA) were designed as described ^[^ ^69^ ^]^ and synthesized (Table S2, Supporting Information). A 21 nt‐long negative control sequence (NC) was used as a control. Fragments harboring *Fto*, *Runx1*, *Fto* shRNA, *scrambled* shRNA, *Fto* gRNA, and NC were ligated into pro‐viral plasmids using the BspEI/NotI, XbaI/NotI, BamHI/XbaI, and BsaI restriction sites, respectively. The resulting vectors expressed the genes under the control of the cytomegalovirus promotor. AAV5 viral particles carrying the cDNA were produced. AAV5‐*Gfp* and AAV5‐*Cre* were purchased from UNC Vector Core. Rat *Fto* siRNA (Catalog number: Sl01571339) and negative control scrambled siRNA (catalog number: Sl03650325) were purchased from Dharmacon, Inc. (Chicago, IL) and *Runx1* siRNA (catalog number: s132294) and *Ehmt2* siRNA (s168026) from Thermo Fisher Scientific, Inc. (Waltham, MA).

##### RNA Immunoprecipitation Assay

The RNA Immunoprecipitation (RIP) assay was conducted using the Magna RIP Kit or the Magna MeRIP m6A Kit (Upstate/EMD Millipore, Darmstadt, Germany). The homogenates from rat DRGs were suspended in the RIP lysis buffer containing protease inhibitor cocktail and RNase inhibitor. The RIP lysate was incubated on ice for 5 min and stored at −80 °C. Magnetic Beads Protein A/G suspension for each IP was washed twice with the RIP wash buffer. Rabbit anti‐FTO antibody (2.0 µg; Santa Cruz), rabbit anti‐m6A antibody (0.6 µg; Millipore), or normal rabbit IgG were conjugated to Magnetic Beads Protein A/G re‐suspended in RIP wash buffer for 30 min at room temperature. After being washed three times with RIP wash buffer, the Beads Protein A/G‐ antibody complexes were re‐suspended into the RIP immunoprecipitation buffer. After being thawed and centrifuged at 14000 rpm at 4 °C for 10 min, the supernatants of the RIP lysate were incubated with beads–antibody complex in the RIP immunoprecipitation buffer overnight at 4 °C by rotating. After the samples were washed six times with the RIP wash buffer, RNA was eluted from the beads by incubating in the proteinase K buffer at 55 °C for 30 min by shaking, purified by phenol/chloroform extraction, and analyzed by RT‐qPCR. The supernatant of the RIP lysate was used as input. All primers used are listed in Table S2, Supporting Information.

##### ChIP Assay

The ChIP assays were carried out using the EZ ChIP Kit (Upstate/EMD Millipore) as described.^[^ ^38^ ^]^ The homogenate from the DRG was crosslinked with 1% formaldehyde at room temperature for 10 min. The reaction was stopped by the addition of glycine (0.25 m). After centrifugation, the pellet was collected and lysed in SDS lysis buffer containing protease inhibitor cocktail. The lysis was sonicated until the DNA was sheared into fragments with a mean length of 200 to 1000 nt. The samples were first pre‐cleaned with protein G magnetic beads and then subjected to immunoprecipitation overnight with 2.5 µg of rabbit anti‐Runx1 antibody or with 2.5 µg of normal rabbit serum overnight at 4 °C. The input (10–20% of the sample for immunoprecipitation) was used as a positive control. After purification, the DNA fragments were amplified using PCR/Real‐time PCR with the primers listed in Table S2, Supporting Information.

##### Luciferase Assay

The 918‐bp fragment from the *Fto* gene promotor region (including Runx1‐binding motif) was amplified by PCR from genomic DNA with the primers (Table S2, Supporting Information) to construct the *Fto* gene reporter plasmid. The PCR products were ligated into the KpnI and HindIII restriction sites of the pGL3‐Basic vector (Promega, Madison, WI). The sequences of recombinant clones were verified by DNA sequencing. PC12 cells were plated on 12‐well plate and cultured at 37 °C in a humidified incubator with 5% CO_2_. One day after culture, the cells of each well were co‐transfected with 300 ng of Runx1 overexpression plasmid, 300 ng of pGL3‐Basic vector with or without the sequences of *Fto* promoter, and 10 ng of the pRL‐TK (Promega) using Lipofectamine 2000 (Invitrogen), according to the manufacturer's instructions. The wells were divided into different groups as indicated. Two days after transfection, the cells were collected and lysed in passive lysis buffer. Approximately 10 µl of supernatant was used to measure the luciferase activity using the Dual‐Luciferase Reporter Assay System (Promega). Independent transfection experiments were repeated three times. The relative reporter activity was calculated after normalization of the firefly activity to renilla activity.

##### m^6^A‐Enhanced Cross‐Linking and Immunoprecipitation‐Sequencing (eCLIP‐seq)

m^6^A‐eCLIP‐seq was conducted as previously reported^[^ ^39^, ^40^ ^]^ with the following modifications: Briefly, 7 days after SNL or sham surgery, ipsilateral rat L5 DRG was collected. Twenty DRGs were pooled together to obtain enough RNA for one repeat. Three repeats were required for each group. Total RNA was extracted and purified as described above. RNA was then fragmented to a size between 50–150 nt by RNA fragmentation reagent (Life Technologies). After the reaction was stopped, the RNA mixtures suspended in CLIP buffer were incubated with anti‐m^6^A antibody (Synaptic System) at 4 °C for 2 h. RNA‐anti‐m^6^A antibody interactions were then stabilized with ultraviolet crosslinking twice in a Stratalinker and precipitated with Protein‐G beads for 2 h at 4 °C, followed by four washes with the lysis buffer. Three 3′‐RNA adaptors (Table S2, Supporting Information) were ligated onto the RNAs from three replicates, respectively, with T4 RNA ligase. Protein–RNA complexes were run on SDS‐PAGE gels, transferred to PVDF membranes. RNAs near the masses of light and heavy chains of antibody were isolated off the membrane. After purification, RNAs were reverse‐transcribed with nested specific primer (Table S2, Supporting Information) as previously reported.^[^ ^70^ ^]^ A 3′‐DNA adaptor was ligated onto the cDNA product (5‐pGNNNNCATAGATCGGAAGAGCGTCG‐3). Libraries were then amplified as previously reported.^[^ ^39^, ^40^ ^]^ PCR primers were designed according to adapters and the commercial indexing and sequencing primers.

Reads from Illumina Hiseq sequencer were analyzed according to previous study.^[^ ^39^, ^40^ ^]^ Briefly, reads were demultiplexed for technical replicates and trimmed with RNA and DNA adaptor sequences. High quality reads were mapped to Rn6 genome along with mRNA splicing information in Rn6 refGene.gtf file. Duplicated reads with identical random sequences were discarded and the unique ones were reserved. m^6^A peaks were picked out using these unique tags. Annotated m^6^A peaks were linked to transcription units. The differential total reads‐normalized tags between SNL and sham groups were calculated. The log2 transformed tag differences between the two groups in intragenic elements were clustered and showed in the heat‐map.

##### Immunohistochemistry

Animals were anesthetized with isoflurane and perfused with 4% paraformaldehyde before being analyzed by single‐ or double‐labeled immunohistochemistry. The L4 and L5 DRGs were collected, post‐fixed, and dehydrated before frozen sectioning at 20 µm. After the sections were blocked for 1 h at room temperature in 0.01 m PBS containing 10% goat serum and 0.3% Triton X‐100, they were incubated with the following primary antibodies or the regents over one or two nights at 4 °C. The antibodies and regents include: mouse anti‐FTO (1:1000, EMD Millipore), mouse anti‐NF200 (1:500, Sigma), mouse anti‐CGRP (1:50, Abcam, Cambridge, MA), rabbit anti‐glutamine synthetase (GS; 1:500, Sigma‐Aldrich), chicken anti‐*β*‐tubulin III (1:1000, Abcam, Cambridge, MA), rabbit anti‐P2X3 (1: 200, Neuromics), rabbit anti‐G9a (1:1000, Abcam, Cambridge, MA), rabbit anti‐MOR (1:1000, Neuromics), rabbit anti‐m^6^A (1:300, Synaptic Systems), mouse anti‐m^6^A (1:300, Synaptic Systems), rabbit anti‐Runx1 (1:100, Abcam), and rabbit anti‐ATF3 (1:200, Sigma). The sections were then incubated with either goat anti‐rabbit antibody conjugated to Cy3 (1:300, Jackson ImmunoResearch, West Grove, PA), and/or goat anti‐mouse or anti‐chicken antibody conjugated to Cy2 (1:300, Jackson ImmunoResearch), for 2 h at room temperature. Control experiments included substitution of normal mouse or rabbit serum for the primary antiserum and omission of the primary antiserum. The sections were finally mounted using VectaMount permanent mounting medium (Vector Laboratories, Burlingame, CA) or Vectashield plus 4′, 6‐diamidino‐2‐phenylindole (DAPI) mounting medium (Vector Laboratories). All immunofluorescence‐labeled images were examined using a Leica DMI4000 fluorescence microscope and captured with a DFC365FX camera (Leica, Germany). Single‐, double‐, or triple‐labeled cells were quantified manually or by using NIH Image J Software.

##### Western Blotting

Bilateral DRGs (L4/5 in rats and L3/4 in mice) and ipsilateral L5 spinal cord were collected. To achieve enough proteins, four unilateral mouse DRGs or two unilateral rat DRGs were pooled together. The tissues were homogenized and the cultured cells ultrasonicated with ice‐cold lysis buffer (10 mm Tris, 2 mm MgCl_2_, 5 mm EGTA, 1 mm EDTA, 1 mm phenylmethylsulfonyl fluoride, 1 mm DTT, 40 µm leupeptin, and 250 mm sucrose). After the crude homogenate was centrifuged at 4 °C for 15 min at 1000 × *g*, the supernatants were collected for cytosolic proteins and the pellets for nuclear proteins. After measuring protein concentration, the samples were heated for 5 min at 99 °C and loaded onto a 4% stacking/7.5% separating SDS‐polyacrylamide gel (Bio‐Rad Laboratories). The proteins were then electrophoretically transferred onto a nitrocellulose membrane (Bio‐Rad Laboratories). After being blocked with 3% nonfat milk in Tris‐buffered saline containing 0.1% Tween‐20 for 1 h, the membranes were then incubated with following primary antibodies overnight: These antibodies included mouse anti‐Kv1.4 (1:500, NeuroMab, Davis, CA), rabbit anti‐GAPDH (1:1000, Santa Cruz, Dallas, TX), mouse anti‐*α*‐tublin (1:1000, Santa Cruz, Dallas, TX), mouse anti‐FTO (1:1000, Abcam), rabbit anti‐G9a (1:1000, Abcam, Cambridge, MA), rabbit anti‐MOR (1:1000, Immunostar), rabbit anti‐Runx1 (1:100, Abcam), rabbit anti‐phospho‐ERK1/2 (Thr202/Tyr204, 1:1000, Cell Signaling), rabbit anti‐ERK1/2 (1:1000, Cell Signaling), mouse anti‐GFAP (1:1000, Cell Signaling), rabbit anti‐histone H3 (1:1000, Cell Signaling), rabbit anti‐DNMT1 (1:1000, Cell Signaling), rabbit anti‐DNMT3a (1:500, Cell Signaling), rabbit anti‐METTL3 (1:1000, Sigma), rabbit anti‐METTL14 (1:1000, LSBio), rabbit anti‐ALKBH5 (1:1000, Proteintech), rabbit anti‐WTAP (1:250, EMD Millipore), and rabbit anti‐YTHDF2 (1:1000, Proteintech). The proteins were detected by horseradish peroxidase‐conjugated anti‐mouse or anti‐rabbit secondary antibody (1:3000, Jackson ImmunoResearch), visualized by western peroxide reagent and luminol/enhancer reagent (Clarity Western ECL Substrate, Bio‐Rad), and exposed using the ChemiDoc XRS System with Image Lab software (Bio‐Rad). The intensity of blots was quantified with densitometry using Image Lab software (Bio‐Rad). The average blot density from the control/naïve (0 day) groups was set as 100%. The relative density values from time points or the treated groups were determined by dividing the optical density values from these groups by the average value of the control/naïve groups after each was normalized to the corresponding histone H3 (for nucleus proteins), GAPDH, or *α*‐tubulin (for cytosolic proteins).

##### Whole‐Cell Patch Clamp Recording

Adult rats spinal cord slices were prepared as described.^[^ ^38^, ^42^ ^]^ Briefly, rats (>28 days) were deeply anesthetized with isoflurane. Laminectomy was applied from mid thoracic to lower lumbar levels. The spinal cord was quickly removed and placed in cold modified artificial cerebrospinal fluid (ACSF) containing (in mm) 80 NaCl, 2.5 KCl, 1.25 NaH_2_PO_4_, 0.5 CaCl_2_, 3.5 MgCl_2_, 25 NaHCO_3_, 75 sucrose, 1.3 ascorbate, 3.0 sodium pyruvate, and oxygenated with 95% O_2_ and 5% CO_2_ (pH 7.4, 310–320 mOsm). Transverse 450 mm slices for miniature EPSC (mEPSC) recording or 500–650 mm slices with attached L4 dorsal root for evoked EPSC (eEPSC) recording were cut by a vibratome VT‐1200 (Leica). Slices were incubated at least 1 h at room temperature in the recording solution containing (in mm) 125 NaCl, 2.5 KCl, 2 CaCl_2_, 1 MgCl_2_, 1.25 NaH_2_PO_4_, 26 NaHCO_3_, 25 d‐glucose, 1.3 ascorbate, 3.0 sodium pyruvate, and oxygenated with 95% O_2_ and 5% CO_2_ (pH 7.4, 310–320 mOsm). Whole‐cell patch recording was carried out as described.^[^ ^38^, ^42^ ^]^ In brief, the slice was transferred to a recording chamber (Warner Instruments, Hamden, CT) and perfused with oxygenated recording solution at a rate of 3 mL min^−1^ at room temperature. Neurons were identified by infrared differential interference contrast (IR‐DIC) with an upright microscope DM6000 (Leica) equipped with a 40 × 0.80 NA water‐immersion objective and a CCD camera (Leica). Whole‐cell patch clamp recording was carried out in the voltage‐clamp mode. The electrode resistances of micropipettes ranged from 3 to 8 MΩ. Lamina II neurons were clamped with an Axopatch‐700B amplifier (Molecular Devices, Downingtown, PA). The intracellular pipette solution contained (in mm) 110 Cs_2_SO_4_, 5 TEA‐Cl, 0.5 CaCl_2_, 2 MgCl_2_, 5 EGTA, 5 HEPES, 5 TEA, 5 Mg‐ATP, 0.5 Na‐GTP, and 1 GDP‐*β*‐S. Cs and TEA were used as a K^+^‐channel blocker and GDP‐*β*‐S used as a GTP binding protein blocker to prevent the postsynaptic µ‐opioid mediated effect as previously described.^[^ ^71^, ^72^ ^]^ To record mEPSC, 500 nm TTX was presented at the recording solution as described above. To record eEPSC, a suction electrode (A‐M systems) was used for electrical stimulation of the attached dorsal root. Stimulation was delivered by the suction electrode with a constant current stimulator S88 (Grass, Rockland, MA). To verify different primary afferent inputs, A*β* fiber‐evoked EPSCs were classified as monosynaptic by constant latency and absence of failures upon 25 µA/20 Hz stimulation; A*δ* fiber‐evoked EPSCs were classified as monosynaptic by constant latency and absence of failures upon 100 µA/2 Hz stimulation; C fiber‐evoked EPSCs were classified as monosynaptic by absence of failures upon 500 µA/1 Hz stimulation. The conduction velocity was further measured to distinguish the primary afferent input (C < 0.8 m s^−1^).^[^ ^73^, ^74^, ^75^ ^]^ Only C fiber input monosynaptic neurons were measured for further experiments. Measurements were made from only one neuron per slice. The pair pulse protocol was delivered by S88 with 20 Hz frequency and 50 ms pulse interval. The signals were filtered at 2 k Hz. Data were stored on computer by a DigiData 1500 interface and were analyzed by the pCLAMP 10.4 software package (Molecular Devices). All experiments were performed at room temperature.

##### Statistical Analysis

For in vitro experiments, the cells were evenly suspended and then randomly distributed in each well tested. For in vivo experiments, the animals were distributed into various treatment groups randomly. All of the results are given as means ± SEM. The data were statistically analyzed with two‐tailed, unpaired Student's *t* test and a one‐way or two‐way ANOVA with repeated measures. When ANOVA showed significant differences, pairwise comparisons between means were tested by the post hoc Tukey method (SigmaStat, San Jose, CA). The sample sizes were determined based on the pilot studies, previous reports in the field, ^[^ ^32^, ^38^, ^61^, ^62^, ^67^ ^]^ and power analyses (power of 0.90 at *p* < 0.05). Significance was set at *p* < 0.05.

## Conflict of Interest

The authors declare no conflict of interest.

## Author Contributions

Y.L., X.G., L.S., J.X., S.S., and S.D. contributed equally to this work. Y.‐X.T. as the primary and corresponding author conceived the project, supervised all experiments, and funded the work. Y.L., X.G., L.S., J.X., S.S., S.D., Z.L., and S.W. contributed toward the development and execution of the project, each making substantial contributions toward this work, including design, acquisition, analysis, or interpretation of the data presented. Y.L. used gRNA strategy to show the role of FTO in SNL and CCI rat models as well as in morphine analgesia and tolerance, double labeled FTO with G9a and m^6^A in DRG neurons, co‐worked with S.W. to show the interaction of Runx1 with *Fto* promoter in DRG of naive and SNL rats, co‐worked with J.X. to reveal the binding of *Ehmt2* mRNA with m^6^A and FTO and wrote the draft of manuscript. X.G. demonstrated Runx1 regulation of FTO in in vitro cell line and in vivo DRG neurons, used FTO transgenic mice to reveal the role of FTO in SNL mouse model, co‐worked with J.X. to show the interaction of YTHDF2 with *Ehmt2* mRNA, demonstrated the involvement of FTO in *Ehmt2* mRNA stabilization, analyzed the bioinformatics data of eCLIP‐seq under the guidance of Y.‐J.C. and prepared all final figures. Both Y.L. and X.G. co‐worked with Z.L. finished patch clamp recording in spinal cord slice. L.S. measured the expression and distribution of FTO in the DRG of naive and model rats, used FTO knockdown (shRNA) and overexpression strategies to show the role of DRG FTO in SNL rat model, collected the tissues to observe the potential downstream targets, and designed/constructed gRNA vectors under the guidance of S.W.. S.S. did SNL model and tissue collection for eCLIP‐seq. S.W. also designed/constructed full length FTO vector, FTO shRNA vector, full length Runx1 vector and corresponding control vectors. K.M. performed some Western blots and quantitative RT‐PCR. S.D. co‐worked with W.L. and S.X. to perform all required experiments for revision. D.D. and Y.‐X.T. wrote/edited the final manuscript. All authors read and discussed the manuscript. Note: The equal contribution statement was added and Figure 4b was updated on 8 July 2020 after original online publication. In Figure 4b, the second column label of each sample was corrected from “CCI” to “SNL.”

## Supporting information

Supporting Information
