# Supplementary material for: N6‐Methyladenosine Demethylase FTO Contributes to Neuropathic Pain by Stabilizing G9a Expression in Primary Sensory Neurons
Source: Adv Sci (Weinh). 2020 May 27;7(13):1902402. doi: 10.1002/advs.201902402 (PMC7341103; doi:10.1002/advs.201902402)
Supplement: Supplementary file 1 — Supporting Information [file ADVS-7-1902402-s001.pdf]

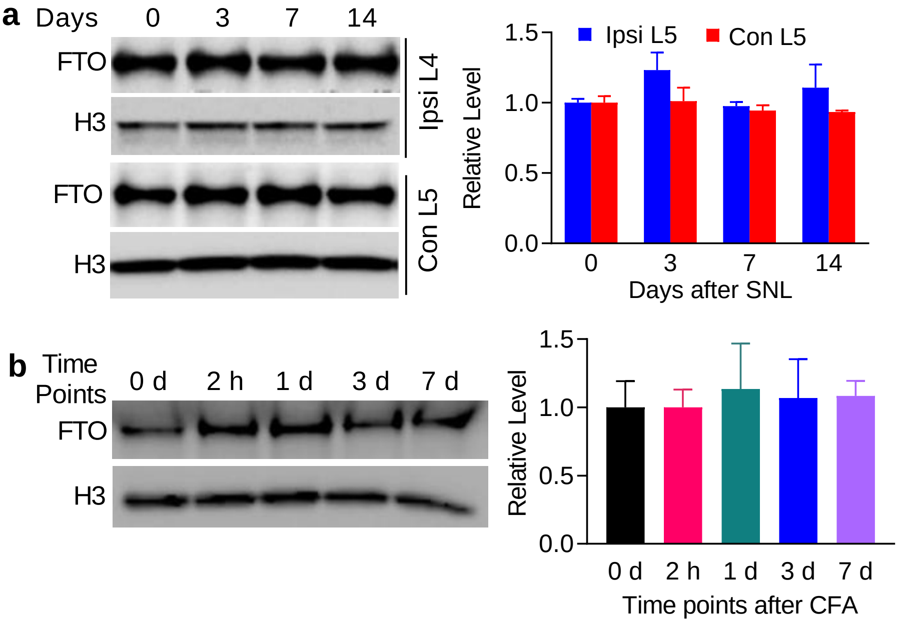
a
Days 0 3 7 14
FTO
H3
FTO
H3
Ipsi L4
Con L5
Ipsi L5
Con L5
Relative Level
1.5
1.0
0.5
0.0
0 3 7 14
Days after SNL
b
Time Points
0 d 2 h 1 d 3 d 7 d
FTO
H3
Relative Level
1.5
1.0
0.5
0.0
0 d 2 h 1 d 3 d 7 d
Time points after CFA

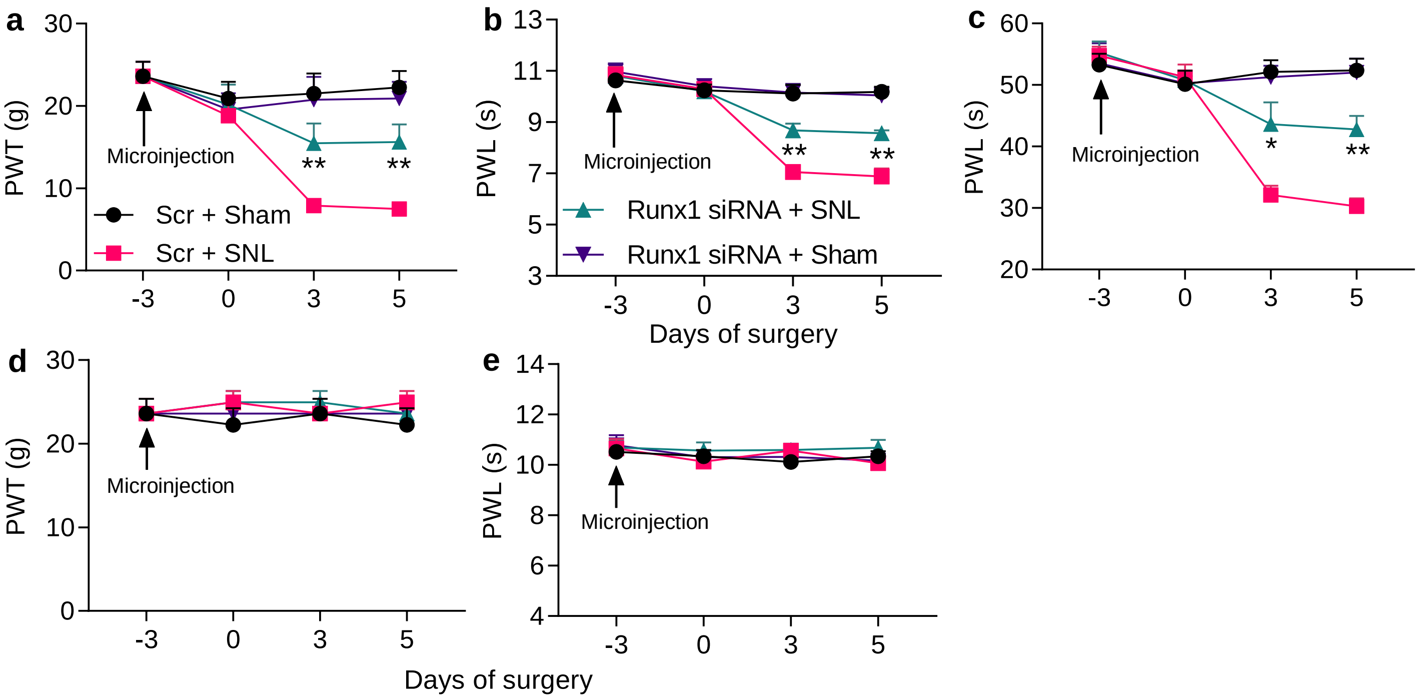

a
b
c
d
e
PWT (g)
PWL (s)
Microinjection
Scr + Sham
Scr + SNL
Runx1 siRNA + SNL
Runx1 siRNA + Sham
Days of surgery
**
*
-3
0
3
5

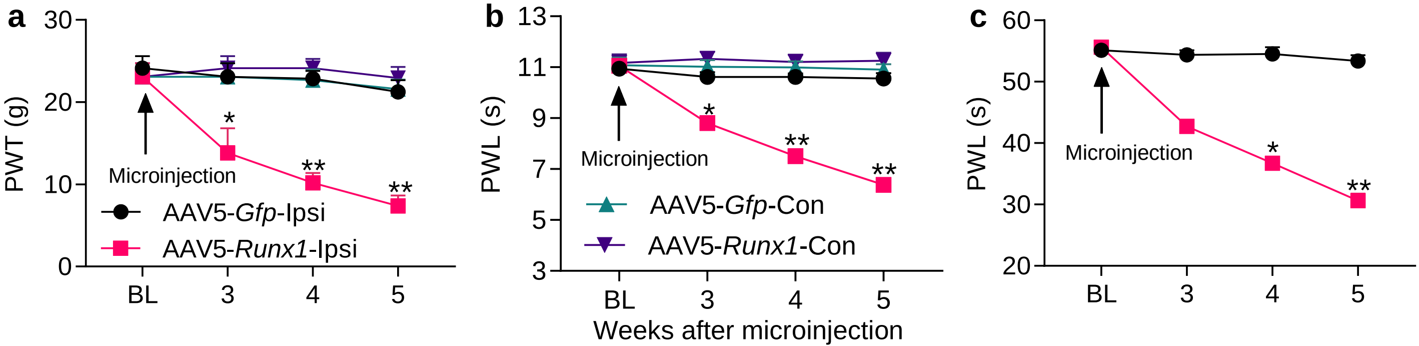
a
b
c
PWT (g)
PWL (s)
PWL (s)
Microinjection
Microinjection
Microinjection
AAV5-*Gfp*-Ipsi
AAV5-*Runx1*-Ipsi
AAV5-*Gfp*-Con
AAV5-*Runx1*-Con
BL
3
4
5
Weeks after microinjection

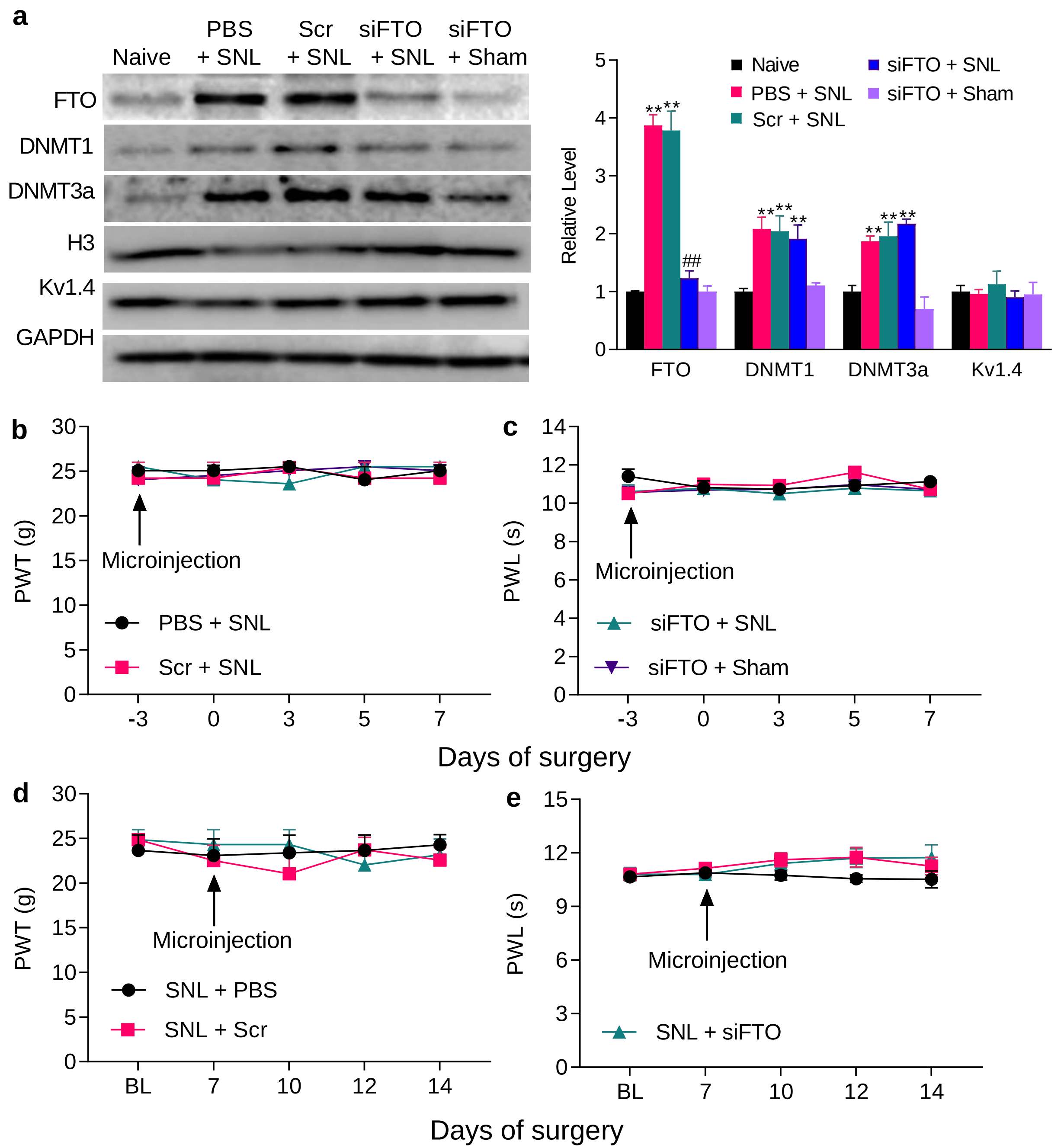
a
Naive
PBS + SNL
Scr + SNL
siFTO + SNL
siFTO + Sham
FTO
DNMT1
DNMT3a
H3
Kv1.4
GAPDH
Relative Level
Naive
PBS + SNL
Scr + SNL
siFTO + SNL
siFTO + Sham
FTO
DNMT1
DNMT3a
Kv1.4
b
PWT (g)
Microinjection
PBS + SNL
Scr + SNL
c
PWL (s)
Microinjection
siFTO + SNL
siFTO + Sham
Days of surgery
d
PWT (g)
Microinjection
SNL + PBS
SNL + Scr
e
PWL (s)
Microinjection
SNL + siFTO
Days of surgery

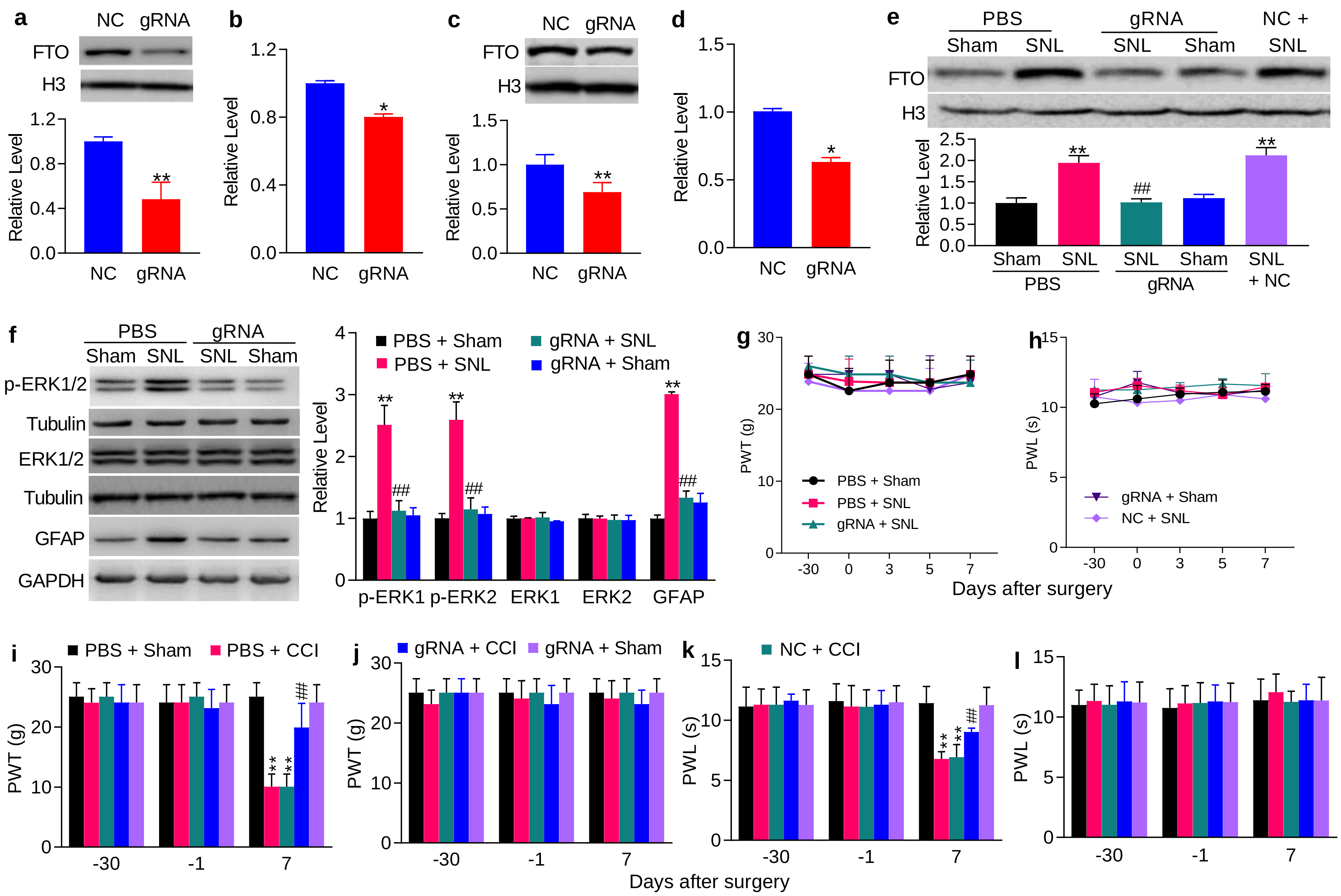
a
NC gRNA
FTO
H3
Relative Level
NC gRNA
b
Relative Level
NC gRNA
c
NC gRNA
FTO
H3
Relative Level
NC gRNA
d
Relative Level
NC gRNA
e
PBS gRNA NC +
Sham SNL SNL Sham SNL
FTO
H3
Relative Level
Sham SNL SNL Sham SNL
PBS gRNA + NC
f
PBS gRNA
Sham SNL SNL Sham
p-ERK1/2
Tubulin
ERK1/2
Tubulin
GFAP
GAPDH
PBS + Sham
PBS + SNL
gRNA + SNL
gRNA + Sham
Relative Level
p-ERK1 p-ERK2 ERK1 ERK2 GFAP
g
PWT (g)
PBS + Sham
PBS + SNL
gRNA + SNL
h
PWL (s)
gRNA + Sham
NC + SNL
Days after surgery
i
PBS + Sham
PBS + CCI
PWT (g)
j
gRNA + CCI
gRNA + Sham
PWT (g)
k
NC + CCI
PWL (s)
l
PWL (s)
Days after surgery

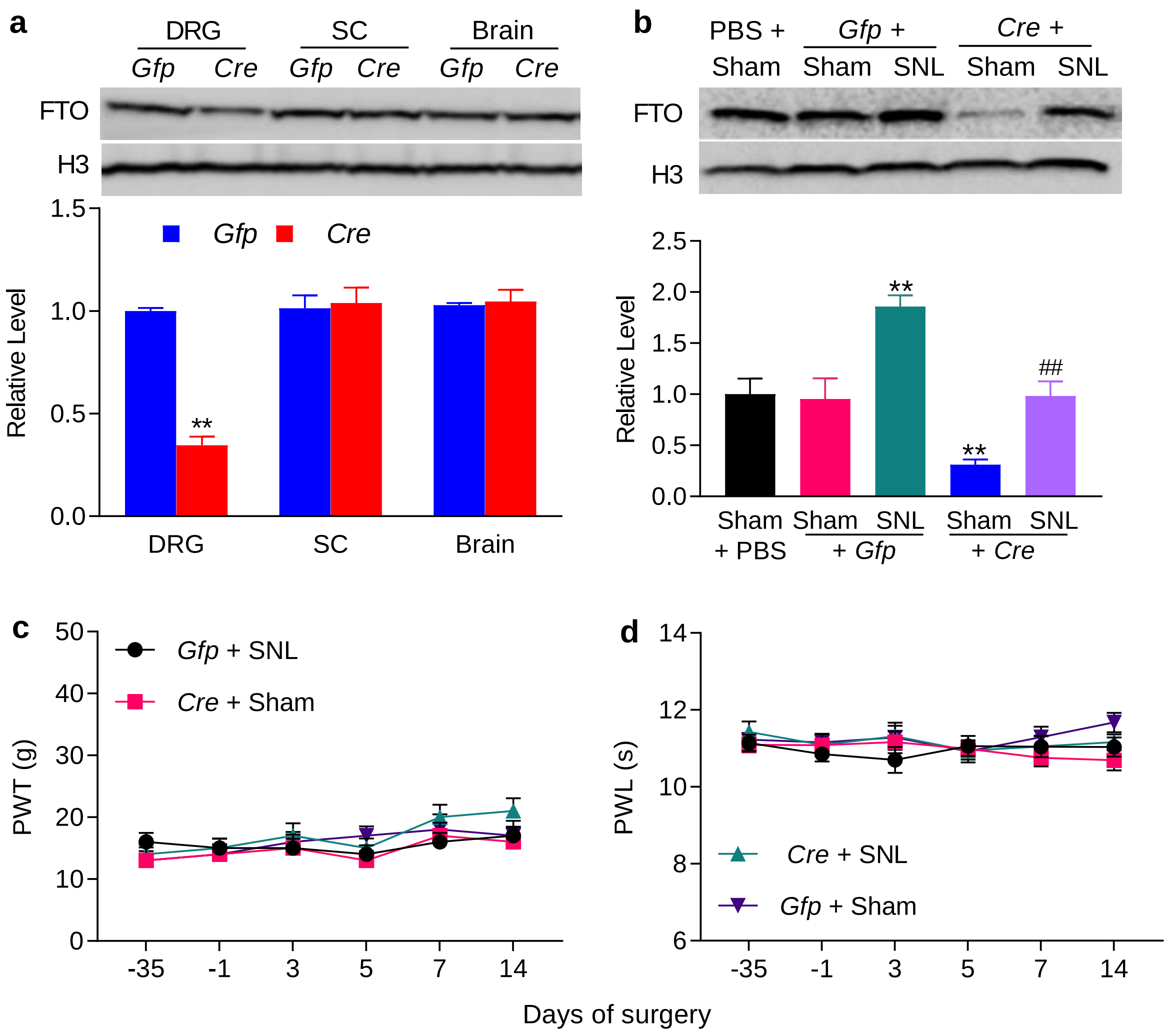
a
DRG
SC
Brain
Gfp Cre Gfp Cre Gfp Cre
FTO
H3
Relative Level
Gfp Cre
**
DRG
SC
Brain
b
PBS + Sham
Gfp + Sham SNL
Cre + Sham SNL
FTO
H3
Relative Level
**
##
Sham + PBS
Sham SNL + Gfp
Sham SNL + Cre
c
PWT (g)
Gfp + SNL
Cre + Sham
d
PWL (s)
Cre + SNL
Gfp + Sham
-35 -1 3 5 7 14
Days of surgery

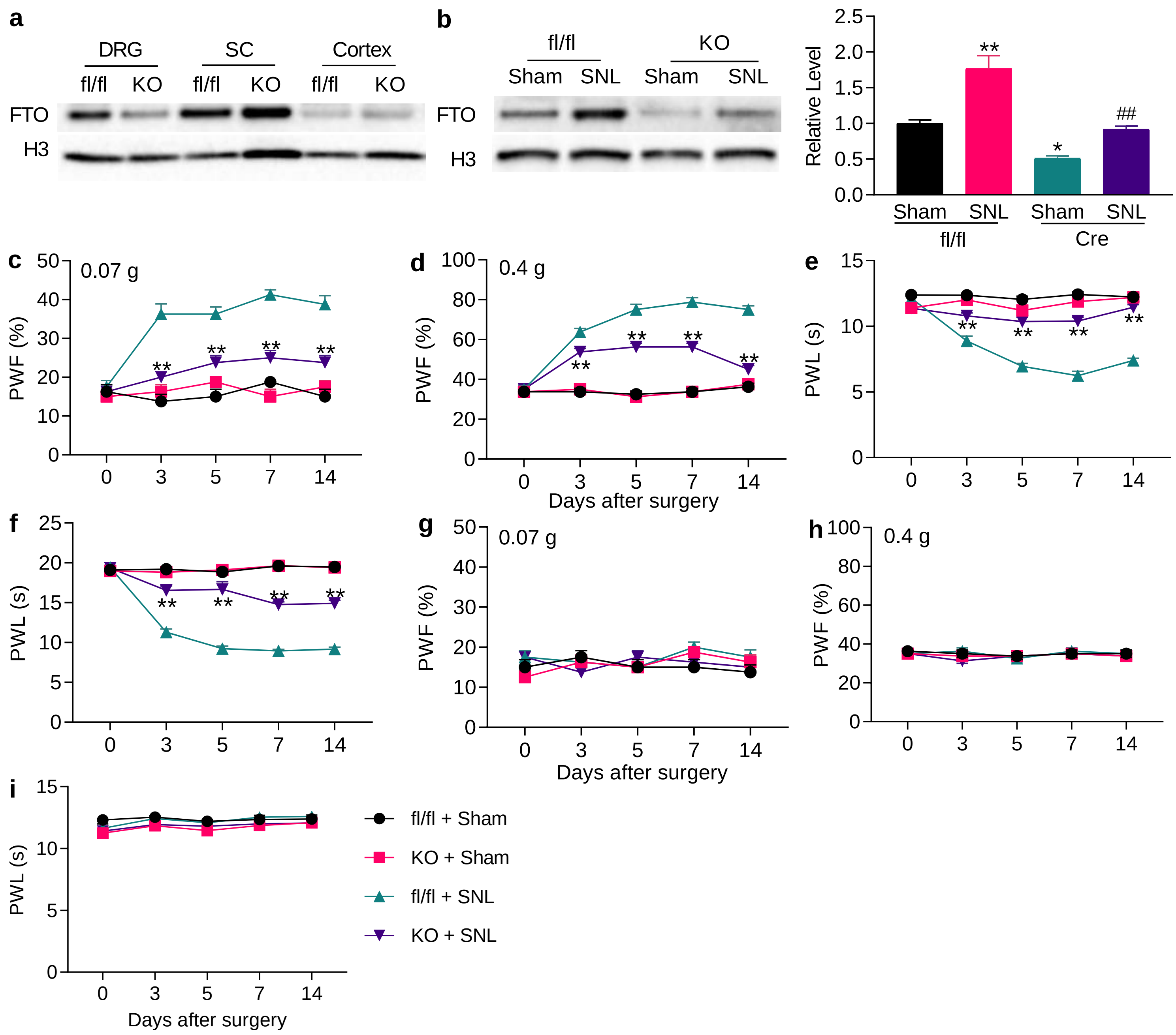

a
DRG
SC
Cortex
fl/fl
KO
fl/fl
KO
fl/fl
KO
FTO
H3
b
fl/fl
KO
Sham
SNL
Sham
SNL
FTO
H3
Relative Level
2.5
2.0
1.5
1.0
0.5
0.0
**
*
##
Sham
SNL
Sham
SNL
fl/fl
Cre
c
0.07 g
PWF (%)
d
0.4 g
PWF (%)
Days after surgery
e
PWL (s)
f
PWL (s)
g
0.07 g
PWF (%)
Days after surgery
h
0.4 g
PWF (%)
i
PWL (s)
Days after surgery
0
3
5
7
14
fl/fl + Sham
KO + Sham
fl/fl + SNL
KO + SNL

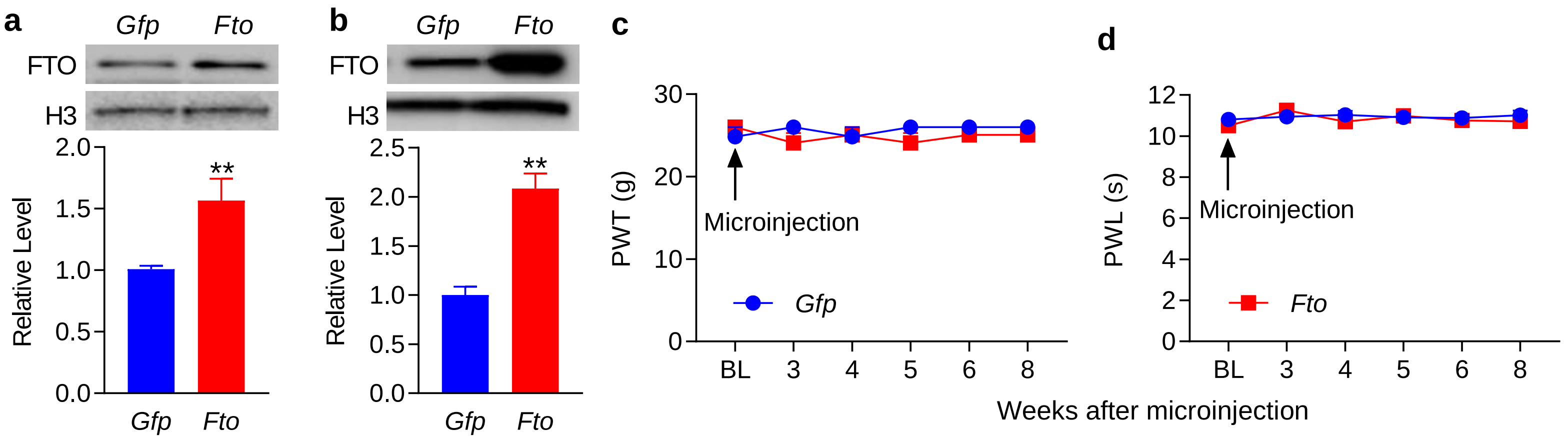
a
Gfp
Fto
FTO
H3
Relative Level
2.0
1.5
1.0
0.5
0.0
**
Gfp
Fto
b
Gfp
Fto
FTO
H3
Relative Level
2.5
2.0
1.5
1.0
0.5
0.0
**
Gfp
Fto
c
PWT (g)
30
20
10
0
Microinjection
Gfp
BL
3
4
5
6
8
d
PWL (s)
12
10
8
6
4
2
0
Microinjection
Fto
BL
3
4
5
6
8
Weeks after microinjection

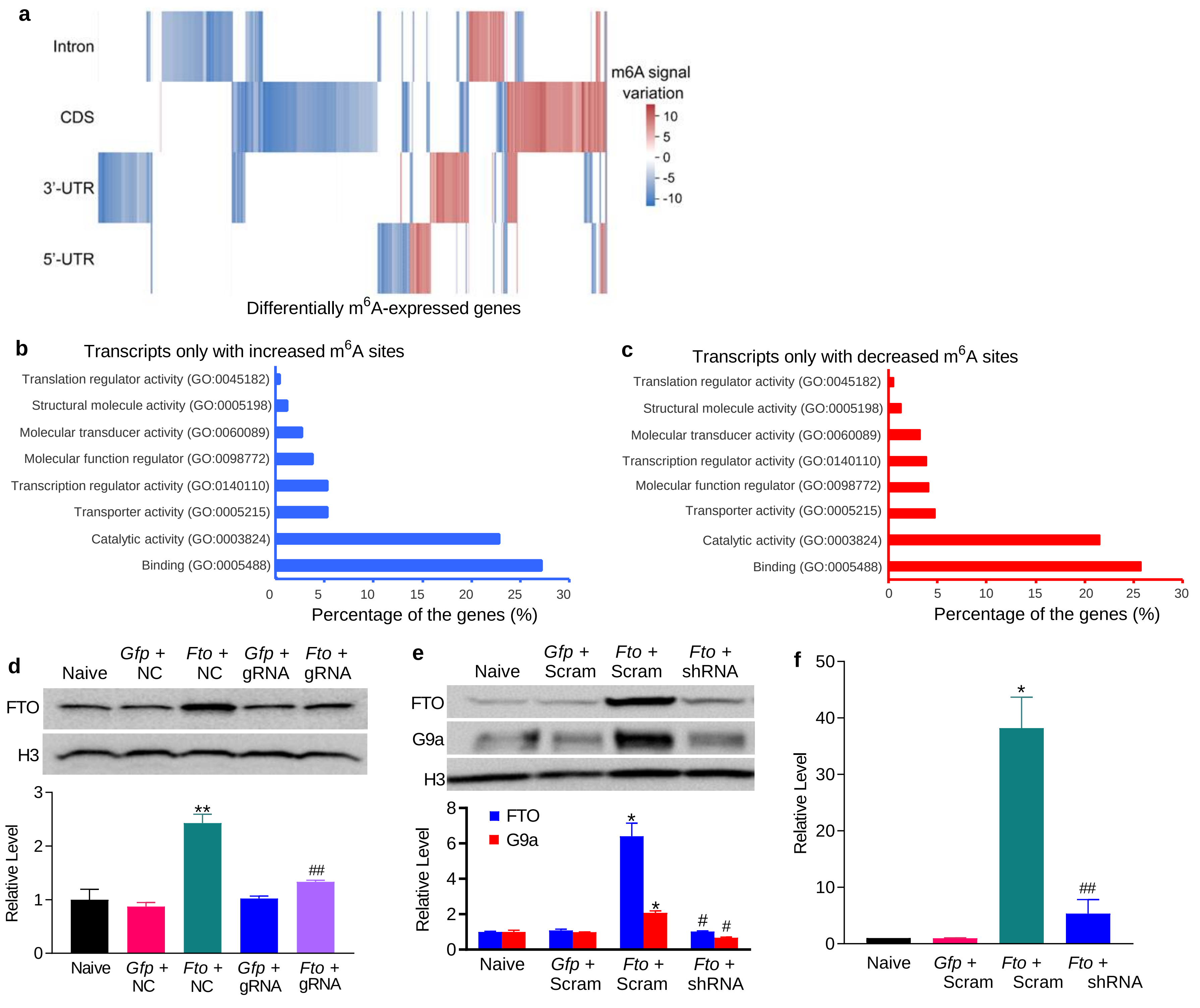

a
Intron
CDS
3'-UTR
5'-UTR
m6A signal variation
10
5
0
-5
-10
Differentially m6A-expressed genes
b
Transcripts only with increased m6A sites
Translation regulator activity (GO:0045182)
Structural molecule activity (GO:0005198)
Molecular transducer activity (GO:0060089)
Molecular function regulator (GO:0098772)
Transcription regulator activity (GO:0140110)
Transporter activity (GO:0005215)
Catalytic activity (GO:0003824)
Binding (GO:0005488)
Percentage of the genes (%)
c
Transcripts only with decreased m6A sites
Translation regulator activity (GO:0045182)
Structural molecule activity (GO:0005198)
Molecular transducer activity (GO:0060089)
Transcription regulator activity (GO:0140110)
Molecular function regulator (GO:0098772)
Transporter activity (GO:0005215)
Catalytic activity (GO:0003824)
Binding (GO:0005488)
Percentage of the genes (%)
d
Naive
Gfp + NC
Fto + NC
Gfp + gRNA
Fto + gRNA
FTO
H3
Relative Level
**
##
e
Naive
Gfp + Scram
Fto + Scram
Fto + shRNA
FTO
G9a
H3
Relative Level
FTO
G9a
*
*
#
#
f
Relative Level
*
##
Naive
Gfp + Scram
Fto + Scram
Fto + shRNA

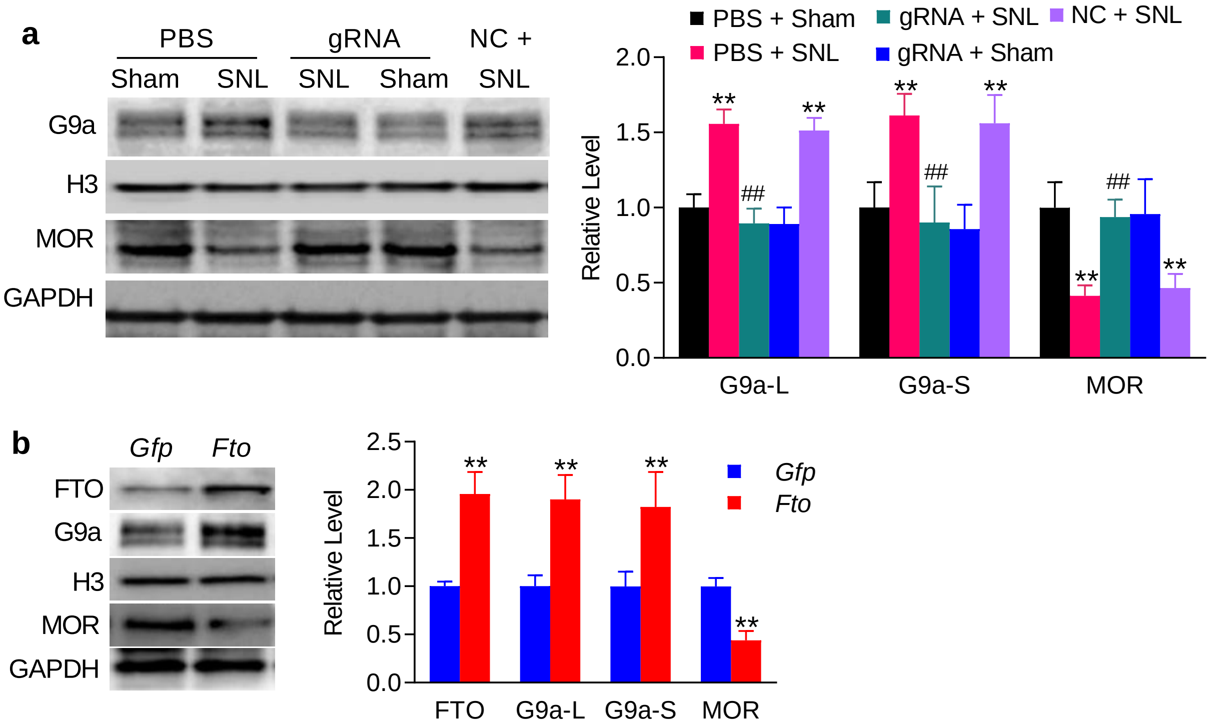

a
PBS
gRNA
NC +
Sham
SNL
SNL
Sham
SNL
G9a
H3
MOR
GAPDH
PBS + Sham
PBS + SNL
gRNA + SNL
gRNA + Sham
NC + SNL
Relative Level
2.0
1.5
1.0
0.5
0.0
**
##
G9a-L
G9a-S
MOR
b
Gfp
Fto
FTO
G9a
H3
MOR
GAPDH
2.5
Relative Level
FTO
G9a-L
G9a-S
MOR

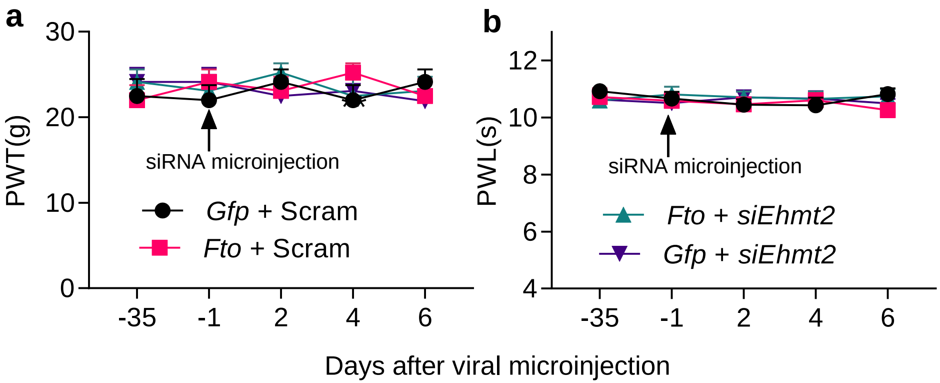

a
30
20
10
0
PWT(g)
siRNA microinjection
Gfp + Scram
Fto + Scram
-35
-1
2
4
6
b
12
10
8
6
4
PWL(s)
siRNA microinjection
Fto + siEhmt2
Gfp + siEhmt2
-35
-1
2
4
6
Days after viral microinjection

Supplementary Figure 1 (**a**) Expression of FTO in the ipsilateral (Ipsi) L4 DRG and contralateral (Con) L5 DRG after SNL. n = 6 rats/time point. One-way ANOVA with repeated measures followed by *post hoc* Tukey test. (**b**) Expression of FTO in the ipsilateral L4/5 DRGs after injection of complete Freund's adjuvant (CFA) into unilateral hind paw. n = 6 rats/time point. One-way ANOVA with repeated measures followed by *post hoc* Tukey test.

Supplementary Figure 2 Effect of pre-microinjection of Runnx1 siRNA or scrambled siRNA (Scr) into the ipsilateral L5 DRG of the rats on the development of SNL-induced mechanical allodynia (**a**), heat hyperalgesia (**b**) and cold allodynia (**c**) on days 3 and 5 post-surgery. (**a**,**d**) Paw withdrawal thresholds (PWT) in response mechanical stimulation on the ipsilateral (**a**) and contralateral (**d**) sides. (**b,c,e**) Paw withdrawal latencies in response to heat stimulation in the ipsilateral (**b**) and contralateral (**e**) and to cold stimulation on the ipsilateral side (**c**). n = 8 rats/group. *$P < 0.05$, **$P < 0.01$ versus the Scr plus SNL group at the corresponding time points by two-way ANOVA with repeated measures followed by *post hoc* Tukey test.

Supplementary Figure 3 Effect of microinjection of AAV5-*Runx1* or AAV5-*Gfp* into unilateral L4/5 DRGs of the rats on paw withdrawal thresholds (PWT) in response to mechanical stimulation (**a**) and on paw withdrawal latencies (PWL) in response to heat (**b**) and cold (**c**) stimuli on the ipsilateral (Ipsi) and contralateral (Con) sides at time points as shown. BL: baseline. n = 10 rats/group. *$P < 0.05$, **$P < 0.01$ versus the AAV5-*Runx1*-treated group on the ipsilateral side at the corresponding time points by two-way ANOVA with repeated measures followed by *post hoc* Tukey test.

Supplementary Figure 4 (**a**) Effect of pre-microinjection of *Fto* siRNA (siFTO), scrambled siRNA (Scr) or PBS into the ipsilateral L5 DRG of the rats on the expression of FTO, DNMT1, DNMT3a and Kv1.4 in the ipsilateral L5 DRG on day 7 after SNL or sham surgery. n = 6

rats/group. One-way ANOVA with repeated measures followed by *post hoc* Tukey test. $**P < 0.01$ versus the corresponding naive group. $##P < 0.01$ versus the corresponding PBS-treated SNL group. (**b,c**) Effect of pre-microinjection of *Fto* siRNA (siFTO), scrambled siRNA (Scr) or PBS into the ipsilateral L5 DRG of the rats on basal paw withdrawal responses to mechanical (**b**) and heat (**c**) stimuli on the contralateral side at days indicated post-SNL or sham surgery. n = 6 rats/group. Two-way ANOVA with repeated measures followed by *post hoc* Tukey test. (**d,e**) Effect of post-microinjection of *Fto* siRNA (siFTO), scrambled siRNA (Scr) or PBS into ipsilateral L5 DRG of the rats on basal paw withdrawal responses to mechanical (**d**) and heat (**e**) stimuli on the contralateral side at days indicated post-SNL or sham surgery. n = 6 rats/group. Two-way ANOVA with repeated measures followed by *post hoc* Tukey test.

Supplementary Figure 5 (**a,b**) Levels of FTO protein (**a**) and mRNA (**b**) in PC12 cells transfected with the *Fto* guide RNA (gRNA) or negative control sequence (NC). n = 3 biological repeats $*P < 0.05$, $**P < 0.01$ versus the NC-treated group by two-tailed unpaired Student's t-test. (**c,d**) Levels of FTO protein (**c**) and mRNA (**d**) in the cultured rat DRG neurons transduced with AAV5-*Fto* guide RNA (gRNA) or AAV5-negative control sequence (NC). n = 3 biological repeats $*P < 0.05$, $**P < 0.01$ versus the AAV5-negative control sequence-treated group by two-tailed unpaired Student's t-test. (**e**) Expression of FTO protein in the ipsilateral L5 DRG on day 7 post-SNL or sham surgery from the rats microinjected with AAV5-*Fto* guide RNA (gRNA), PBS or AAV5-negative control sequence (NC). n = 3 biological repeats (6 rats)/group. Two-way ANOVA with repeated measures followed by post hoc Tukey test. $**P < 0.01$ versus the PBS-treated sham group. $##P < 0.01$ versus the PBS-treated SNL group. **(f)** Levels of p-ERK1/2, ERK1/2 and GFAP in the ipsilateral L5 dorsal horn 7 days after SNL or sham surgery from the rats microinjected with AAV5-*Fto* guide RNA (gRNA), PBS or AAV5-negative control

sequence (NC). n = 3 biological repeats (3 rats)/group. Two-way ANOVA with repeated measures followed by post hoc Tukey test. $^{**}P < 0.01$ versus the corresponding PBS-treated sham group. ##$P < 0.01$ versus the corresponding PBS-treated SNL group. (**g,h**) Effect of pre-microinjection of AAV5-*Fto* guide RNA (gRNA), PBS or AAV5-negative control sequence (NC) into ipsilateral L5 DRG on basal paw withdrawal responses to mechanical **(g)** and heat **(h)** stimuli on the contralateral side at days indicated post-SNL or sham surgery. PWT: paw withdrawal threshold. PWL: paw withdrawal latency. Two-way ANOVA with repeated measures followed by post hoc turkey test. n = 5 rats/group. (**i-l**) Effect of pre-microinjection of AAV5-*Fto* guide RNA (gRNA), PBS or AAV5-negative control sequence (NC) into ipsilateral L4/5 DRGs on paw withdrawal thresholds (PWT) in response to mechanical stimulation **(i, j)** and paw withdrawal latencies (PWL) in response to heat stimulation **(k, l)** stimuli on the ipsilateral (**i, k**) and contralateral (**j, l**) sides at days indicated post-CCI or sham surgery. Two-way ANOVA with repeated measures followed by post hoc turkey test. n = 6 rats/group. $^{**}P < 0.01$ versus the corresponding PBS-treated sham group. ##$P < 0.01$ versus the corresponding PBS-treated CCI group.

Supplementary Figure 6 (**a**) FTO protein expression in the ipsilateral L4 DRG, spinal cord (SC) and brain 7 weeks after microinjection of AAV5-*Gfp* (*Gfp)* or AAV5-*Cre* (*Cre*) into unilateral L4 DRG from the $Fto^{fl/fl}$ mice. n = 3 biological repeats (6 mice). $^{**}P < 0.01$ versus the corresponding AAV5-*Gfp*-treated group by two-tailed unpaired Student's t-test. (**b**) FTO protein expression in the ipsilateral L4 DRG on day 14 post-SNL or sham surgery from the $Fto^{fl/fl}$ mice microinjected with AAV5-*Cre* or AAV5-*Gfp*. n = 3 biological repeats (12 mice)/group. One-way ANOVA followed by post hoc Tukey test. $^{**}P < 0.01$ versus the PBS-treated sham group. ##$P <$ 0.01 versus the AAV5-*Gfp*-treated SNL group. (**c, d**) Effect of pre-microinjection of AAV5-*Gfp*

(*Gfp*) or AAV5-*Cre* (*Cre*) into ipsilateral L4 DRG of the *Fto*$^{fl/fl}$ mice on basal paw withdrawal responses to mechanical **(c)** and heat **(d)** stimuli on the contralateral side at days indicated post-SNL or sham surgery. PWF: paw withdrawal frequency. PWL: paw withdrawal latency. Two-way ANOVA with repeated measures followed by post hoc turkey test. n = 10 mice/group.

Supplementary Figure 7 (**a**) FTO protein expression in the DRGs, spinal cord (SC) and cortex from the *Fto*$^{fl/fl}$ mice (fl/fl) and the conditional *Fto* knockout mice (KO). (**b**) Level of FTO protein in the ipsilateral L4 DRG on day 14 post-SNL or sham surgery from the *Fto*$^{fl/fl}$ mice (fl/fl) and the conditional *Fto* knockout mice (KO). n = 3 biological repeats (12 mice)/group. One-way ANOVA with repeated measures followed by post hoc Tukey test. *$P < 0.05$, **$P < 0.01$ versus the sham *Fto*$^{fl/fl}$ mice. ##$P < 0.01$ versus the SNL conditional *Fto* knockout mice. (**c-i**) Paw withdrawal frequency (PWF) to low (0.07 g; **c** and **g**) and medium (0.4 g; **d** and **h**) force von Frey filament stimuli and paw withdrawal latency (PWL) to heat (**e** and **i**) and cold (**f**) stimuli on the ipsilateral (**c-f**) and contralateral (**g-i**) sides from *Fto*$^{fl/fl}$ mice (fl/fl) or conditional *Fto* knockout mice (KO) after SNL or sham surgery. n = 10 mice/group. **$P < 0.01$ versus the SNL *Fto*$^{fl/fl}$ mice at the corresponding time points by two-way ANOVA with repeated measures followed by post hoc Tukey test.

Supplementary Figure 8 (**a**) FTO protein expression in the cultured rat DRG neurons transduced with AAV5-*Fto* (*Fto*) or AAV5-*Gfp* (*Gfp*). n = 3 biological repeats/group. **$P < 0.01$ versus the AAV5-*Gfp*-treated group by two-tailed unpaired Student's t-test. **(b)** FTO protein expression in the ipsilateral L4/5 DRGs 8 weeks after microinjection of AAV5-*Fto* (*Fto*) or AAV5-*Gfp* (*Gfp*) into unilateral L4/5 DRGs of the rats. n = 3 biological repeats (6 rats)/group. **$P < 0.01$ versus the AAV5-*Gfp*-treated group by two-tailed unpaired Student's t-test. (**c, d**) Basal paw withdrawal response to mechanical (**c**) and heat (**d**) stimuli on the contralateral side

on weeks as indicated after microinjection of AAV5-*Fto* (*Fto*) or AAV5-*Gfp* (*Gfp*) into unilateral L4/5 DRGs of the rats. BL: baseline. PWT: paw withdrawal threshold. PWL: paw withdrawal latency. n = 12 rats/group. Two-way ANOVA with repeated measures followed by post hoc Tukey test.

Supplementary Figure 9 (**a**) Dynamic changes of m^6A sites across transcripts from the ipsilateral L5 DRG on day 7 after SNL or sham surgery. This chart is based on clustering relative tag numbers of peak-associated genes. The intragenic regions of peak-associated genes are classified into four categories: intron, CDS (coding sequence region), 5'-UTR and 3'-UTR. Peaks are categorized into each of the intragenic regions. Thus, each gene has four values according to the relative tag numbers in the four regions. The relative tag numbers are log2_ratio of different tag numbers ((tag number in the SNL group) – (tag number in the sham group)) in peaks in each region. If no peaks were detected in one gene locus in both sham and SNL groups, this gene was discarded for analysis. (**b,c**) GO enrichment analyses of the transcripts with increased (**b**) and decreased (**c**) m^6A sites from the ipsilateral L5 DRG on day 7 after SNL or sham surgery. (**d**) FTO protein expression in cultured rat DRG neurons transduced with the viruses as indicated. *Gfp*: AAV5-*Gfp*. *Fto*: AAV5-*Fto*. gRNA: AAV5-*Fto* guide RNA. NC: AAV5-control negative sequence. n = 3 biological repeats/group. One-way ANOVA with repeated measures followed by post hoc Tukey test. ***P* < 0.01 versus the *Gfp* plus NC-treated group. ##*P* < 0.01 versus the *Fto* plus NC-treated group. (**e,f**) Levels of FTO and G9a proteins (**e**) and *Fto* mRNA (**f**) in PC12 cells transduced by the vectors as shown. *Gfp*: vector expressing *Gfp*. *Fto*: vector expressing full-length *Fto*. Scr: vector expressing scrambled shRNA. shRNA: vector expressing *Fto* shRNA. n = 3 biological repeats. One-way ANOVA with repeated

measures followed by *post hoc* Tukey test. *P < 0.05 versus corresponding naive group. #P < 0.05, ##P < 0.01 versus the corresponding *Fto* plus Scr-treated group.

Supplementary Figure 10 (**a**) Levels of G9a and MOR protein in the ipsilateral L5 DRG on day 7 post-SNL or sham surgery from the rats microinjected with AAV5-*Fto* guide RNA (gRNA), PBS or AAV5-negative control sequence (NC). n = 3 biological repeats (6 rats)/group. One-way ANOVA with repeated measures followed by post hoc Tukey test. **P < 0.01 versus the corresponding PBS-treated sham group. ##P < 0.01 versus the corresponding PBS-treated SNL group. (**b**) Levels of FTO, G9a and MOR proteins in the cultured rat DRG neurons transduced with AAV5-*Fto* (*Fto*) or AAV5-*Gfp* (*Gfp*). n = 3 biological repeats. **P < 0.01 versus the corresponding AAV5-*Gfp*-treated group by two-tailed unpaired Student's t-test.

Supplementary Figure 11 Effect of microinjection of *Ehmt2* siRNA (*siEhmt2*) or scrambled siRNA (Scram) into ipsilateral L4/5 DRGs on contralateral paw withdrawal responses to mechanical (**a**) and heat (**b**) stimuli at time points as shown in the rats pre-microinjected with AAV5-*Fto* (*Fto*) or AAV5-*Gfp* (*Gfp*) into unilateral L4/5 DRGs. PWT: paw withdrawal threshold. PWL: paw withdrawal latency. n = 10 rats/group. Two-way ANOVA with repeated measures followed by *post hoc* Tukey test.

Supplementary Table 1: Locomotor functions

Treatments/genotyping	Functional test		
	Placing	Grasping	Righting
Fto siRNA + Sham	5 (0)	5 (0)	5 (0)
Fto siRNA + SNL	5 (0)	5 (0)	5 (0)
Fto scramble + SNL	5 (0)	5 (0)	5 (0)
PBS + SNL	5 (0)	5 (0)	5 (0)
PBS + Sham	5 (0)	5 (0)	5 (0)
sg3+Sham	5 (0)	5 (0)	5 (0)
sg3+ SNL	5 (0)	5 (0)	5 (0)
NC + Sham	5 (0)	5 (0)	5 (0)
NC + SNL	5 (0)	5 (0)	5 (0)
$Fto^{fl/fl}$ + AAV5-Cre + Sham	5 (0)	5 (0)	5 (0)
$Fto^{fl/fl}$ + AAV5-Cre + SNL	5 (0)	5 (0)	5 (0)
$Fto^{fl/fl}$ + AAV5-GFP + Sham	5 (0)	5 (0)	5 (0)
$Fto^{fl/fl}$ + AAV5-GFP + SNL	5 (0)	5 (0)	5 (0)
AAV5-GFP	5 (0)	5 (0)	5 (0)
AAV5-FTO	5 (0)	5 (0)	5 (0)
$Fto^{fl/fl}$ + Sham	5 (0)	5 (0)	5 (0)
$Fto^{fl/fl}$ + SNL	5 (0)	5 (0)	5 (0)
Adv^{cre} $Fto^{fl/fl}$ + Sham	5 (0)	5 (0)	5 (0)
Adv^{cre} $Fto^{fl/fl}$ + SNL	5 (0)	5 (0)	5 (0)

Scores for placing, grasping and righting reflexes were based on counts of each normal reflex exhibited in five trials. All values are Mean (SEM). n = 10 rats or mice/group.

Supplementary Table 2. All primers and other sequences used.

**PCR or Real-RT-PCR**
Fto-F 5’- TGGATCCGTGCATCTGTAAAGCTCAGG -3’
Fto-R 5’- CCTGACAAATAACGACAATCGAGATG -3’
Ehmt2-F 5’-TGCCTATGTGGTCAGCTCAG-3’
Ehmt2-R 5’-GGTTCTTGCAGCTTCTCCAG-3’
Cre-F 5’-TGC CAC GAC CAA GTG ACA GCA ATG-3’
Cre-R 5’-ACC AGA GAC GGA AAT CCA TCG CTC-3’
Rat-*Fto*-F 5’- TCTTACAACGCTGCCAGTTG -3’
Rat-*Fto*-R 5’- GAAACCAGAACTGCCTCAGC -3’
Mouse-*Fto*-F 5’- GCGGGAAGCTAAGAAACTGA -3’
Mouse-*Fto*-R 5’- ATGCAGCTCCTCTGGTATGC -3’
Rat-*Runx1*-F 5’- CGAAGACATCGGCAGAAACT -3’
Rat-*Runx1*-R 5’- GCTGAGGGTTAAAGGCAGTG -3’
Rat-*Ehmt2*-F 5’- GATCATCTGCCGGGATGTAG -3’
Rat-*Ehmt2*-R 5’- AGTGCTGCAGGTGAGTGATG -3’
Mouse-*Ehmt2*-F 5’- AAATTGGGAACTTGGAAATGG-3’
Mouse-*Ehmt2*-R 5’- CACTACCCGTGAAGGAGGC-3’
Gapdh-F 5’-TCG GTG TGA ACG GAT TTG GC-3’
Gapdh-R 5’-TCC CAT TCT CGG CCT TGA CT-3’
Rat-*Oprm1*-F 5’- TTCCTGGTCATGTATGTGATTGT-3’
Rat-*Oprm1*-R 5’- GGGCAGTGTACTGGTCGCTAA-3’
Rat-*Oprk1*-F 5’- TTTGTGGTGGGCTTAGTGGG -3’
Rat-*Oprk1*-R 5’- CTCTGGAAGGGCATAGTGGT -3’
Kcna2-F 5’- CTGCAAGGGCAACGTCACAC -3’
Kcna2-R 5’-GGGACAGTGAGATGCTTGGC-3’
**ChIP-PCR**
Fto-ChIP-F 5’- GAGTGGCACCACACCCTACT -3’
Fto-ChIP-R 5’- GACAGGGTAGGGGGACGTAT-3’
**RIP-PCR**
Rat Ehmt2-RIP-F 5’- GATCATCTGCCGGGATGTAG-3’
Rat Ehmt2-RIP-R 5’-AGTGCTGCAGGTGAGTGATG-3’
***Fto* Guide RNA**
GATGAGGATGCGAGACACCGGG (PAM: GTGAG, targeting exon 3)
***Fto* shRNAs**
Fto siRNA: ACGUGACUUUGCUAAACUUTT
Fto siRNA: AAGUUUAGCAAAGUCACGUTG
Scramble siRNA: AAGGCUCUAUGAAGAGGCUTG
**Cloning**
FTO shRNA sense: GATCCGATGAAGTGGACCTTAAGAGAAGCTTGTCTTAAGGTCCACTTCATCTTT TTTT
FTO shRNA antisense: CTAGAAAAAAAGATGAAGTGGACCTTAAGACAAGCTTCTCTTAAGGTCCACTTC

ATCG
Runx1 shRNA sense: GATCCGCAGGCTCCTACCAATTCTGAAGCTTGAGAATTGGTAGGAGCCTGCTTTTTTT
Runx1 shRNA antisense: CTAGAAAAAAAGCAGGCTCCTACCAATTCTCAAGCTTCAGAATTGGTAGGAGCCTGCG
Scramble shRNA sense: GATCCGGTTCAGATGTGCGGCGAGTGAAGCTTGACTCGCCGCACATCTGAACCTTTTTTT
Scramble shRNA antisense: CTAGAAAAAAAGGTTCAGATGTGCGGCGAGTCAAGCTTCACTCGCCGCACATCTGAACCG
Runx1 RT F: TAACCCTGCCTGGGTGTAAG
Runx1 RT R: AATAACGACCACCCAGATGC
Runx1 nested F: CGCTCTAGAGCCACCATGCGTATCCCCGTAGA
Runx1 nested R: ATATAGCGGCCGCTCAGTAGGGCCGCCAGACA
Fto RT F: ATGAAGCGCGTCCAGACCGC
Fto RT R: GGGAGAAAAGCCAAGGACAT
Fto nested F: ATATCCGGAGCCACCATGAAGCGCGTCCAGACC
Fto nested R: GCTAGCGGCCGCCTAGGATCTTGCTTCCAG
FTO-luc-F: ATAGGTACCTAAGGGAAGCCTATGCAAGC
FTO-luc-R: ATCAAGCTTACTCAGGCCTGCATCACAG
**m^{6}A-eCLIP-seq**
3’-RNA adaptor 1: ATTGCTT AGATCGGAAGAGCACACGTCT
3’-RNA adaptor 2: ACAAGCCAGATCGGAAGAGCACACGTCT
3’-RNA adaptor 3: AACTTGT AGATCGGAAGAGCACACGTCT
nested specific primer: AGACGTGTGCTCTTCCGA

RT: reverse transcription. F: forward. R: reverse.
